# Chemoenzymatic Synthesis of *D*-Glucitol-Based Non-Ionic Amphiphilic Architectures as Nanocarriers

**DOI:** 10.3390/polym12061421

**Published:** 2020-06-25

**Authors:** Priyanka Manchanda, Katharina Achazi, Diksha Verma, Christoph Böttcher, Rainer Haag, Sunil K. Sharma

**Affiliations:** 1 Department of Chemistry, University of Delhi, Delhi-110 007, India; priyanka23288@gmail.com (P.M.); diksha.17dec@gmail.com (D.V.); 2 Institut für Chemie und Biochemie, Freie Universität Berlin, Arnimallee 22, 14195 Berlin, Germany; katharina.achazi@fu-berlin.de; 3 Forschungszentrum für Elektronenmikroskopie, Institut für Chemie und Biochemie, Freie Universität Berlin, Fabeckstraße 36a, 14195 Berlin, Germany; christoph.boettcher@fzem.fu-berlin.de; 4 Institut für Chemie und Biochemie, Freie Universität Berlin, Takustraße 3, 14195 Berlin, Germany

**Keywords:** *D*-Glucitol, *Candida antarctica* lipase, non-ionic amphiphiles, click chemistry, cyto-compatible, nanocarrier

## Abstract

Newer non-ionic amphiphiles have been synthesized using biocompatible materials and by following a greener approach i.e., *D*-glucitol has been used as a template, and hydrophobic and hydrophilic segments were incorporated on it by using click chemistry. The hydrophilic segments in turn were prepared from glycerol using an immobilized *Candida antarctica* lipase (Novozym-435)-mediated chemoenzymatic approach. Surface tension measurements and dynamic light scattering studies reflect the self-assembling behavior of the synthesized amphiphilic architectures in the aqueous medium. The results from UV-Vis and fluorescence spectroscopy establish the encapsulation of guests in the hydrophobic core of self-assembled amphiphilic architectures. The results of 3-(4,5-dimethylthiazol-2-yl)-5-(3-carboxymethoxyphenyl)-2-(4-sulfophenyl)-2*H*-tetrazolium (MTS) assay indicate that the amphiphiles are well tolerated by the used A549 cell lines at all tested concentrations.

## 1. Introduction

The natural biological constructs such as proteins, lipids, nucleic acids, etc. display self-assembling behavior governed by various non-covalent interactions [1]. These self-assemblies lead to stable and functional supramolecular architectures that form the basis of life [2]. The aspiration to imitate these highly specialized natural self-assemblies has stimulated the recent developments in the field of self-assembly of synthetic amphiphilic architectures as functional nanomaterials [3].

Among the plethora of architectures capable of exhibiting self-assembling properties, low molecular weight amphiphiles, in particular the Gemini amphiphiles, have attracted much attention of researchers, both in academia and industry, due to their superior physicochemical properties as compared to their ionic, in particular the cationic counterparts [4,5,6,7,8,9].

Amphiphiles owing to their bipolar nature self-assemble in an aqueous medium and lead to the creation of an internal cavity in the self-assembled constructs [10]. The driving force behind the formation of self-assemblies lies in their amphiphilic molecular skeleton, where varied non-covalent interactions such as hydrophobic, ionic, hydrogen bonding, and π–π interactions orient them to attain a state of minimum free energy [11]. The internal cavity thus formed is being utilized in addressing the challenges and limitations of drug delivery such as poor aqueous solubility, high drug dosage, and short half-life in bloodstream [12,13,14,15].

**Scheme 1:**
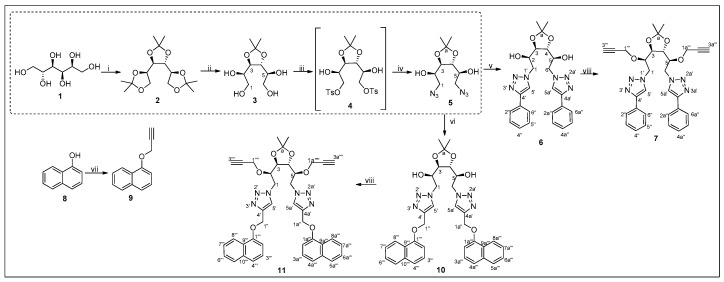
Synthesis of glucitol-3,4-acetonide-1,6-diazide and hydrophobic backbone. (i) acetone, H_2_SO_4_, 18 h, 30 °C; (ii) 70% CH_3_COOH, 35 min, 35 °C; (iii) *p*-toluenesulfonyl chloride, pyridine, 4 h, −5–0 °C; (iv) NaN_3_, Dimethylformamide (DMF), 70 °C, 12 h; (v) C_6_H_5_C≡CH, CuSO_4_∙5H_2_O, sodium ascorbate, THF:H_2_O (3:1); (vi) **9**, [Cu(PPh_3_)_3_]Br, *N*,*N*-diisopropylethylamine (DIPEA), Dichloromethane (DCM), 30 °C, 24 h; (vii) K_2_CO_3,_ propargyl bromide, acetone, 50 °C, 12 h; (viii) NaH, propargyl bromide, THF, 25 °C, 12 h.

However, cytocompatibility is a preliminary requirement for the amphiphilic self-assemblies to be applicable in the biomedical field. Thus, we have opted to use easily available biocompatible material to design and develop non-cytotoxic amphiphilic architectures as nanocarriers. In this endeavor, *D*-glucitol, a carbohydrate, also known as *D*-sorbitol, is used in various diet foods (including diet drinks and ice cream), cough syrups, sugar-free chewing gums, mouthwashes, toothpastes, and cosmetics and has attracted our interest. *D*-Glucitol, comprising of two primary and four secondary hydroxyl groups, can be differently functionalized and explored for the synthesis of varied molecules. *D*-Glucitol-based sorbitan esters (spans)/ethoxylated sorbitan esters (Tween) and others have been extensively employed in various healthcare products, cosmetics, and food industry as emulsifiers, stabilizers, and excipients [16,17,18,19]. Furthermore, glucitol has been employed as the hydrophilic component in the synthesis of amphiphilic constructs, and their gelation behavior has been studied for varied applications [20,21,22,23,24,25,26,27,28]. However, the *D*-glucitol-based amphiphiles as a nanocarrier remains unreported. Herein, we report the synthesis and biophysical characterization of novel non-ionic amphiphilic constructs constituted from glucitol-3,4-monoacetonide-1,6-diazide as a template and conjugating it with biocompatible synthons. Both polar and non-polar components have been grafted on the glucitol backbone via alkyne–azide-based ‘cycloaddition’ reaction (Scheme 1). Triglyceryl azide, methoxypolyethylene glycol azide, and glyceryl azide were employed as hydrophilic moiety and aromatic units such as phenyl or naphthyl used to confer hydrophobicity. Our objective is to investigate the capability of these amphiphiles to form supramolecular assembly to encapsulate guest molecules. The self-assembling behavior of the synthesized amphiphiles in aqueous medium was investigated via dynamic light scattering (DLS), surface tension measurements, and cryogenic electron transmission microscopy (cryo-TEM). Subsequent illustration of the applicability of the synthesized amphiphiles as nanocarriers has been provided by evaluating their cytotoxicity profile in addition to the ability of the self-assembled aggregates to encapsulate hydrophobic guests.

## 2. Materials and Methods

### 2.1. Materials

All commercially available compounds were used as received without further purification. All the chemicals and solvents used were procured from Spectrochem Pvt. Ltd., India, and Sigma-Aldrich Chemicals, USA. Immobilized *Candida antarctica* lipase (Novozym-435) was purchased from Julich Chiral Solutions GmbH (Jülich, Germany). Nile red/dexamethasone used for encapsulation studies were purchased from Fluka Chemie GmbH (Buchs, Switzerland) and Sigma-Aldrich Chemical, USA with maximum purity. The progress of the reactions was monitored using pre-coated TLC plates (Merck silica gel 60F_254_) under a UV lamp, and in other cases, cerric solution was used for visualization of the spots on TLC. Column chromatography was performed using silica gel (100–200 mesh). Benzoylated dialysis tubing (Molecular weight cut-off 2000 Da) used for the purification of amphiphiles by dialysis was procured from Sigma-Aldrich Chemicals, USA. Millipore water used for preparing samples for physicochemical studies, and guest encapsulation was obtained from Milli-Q Integral Water Purification System.

### 2.2. Instrumentation and Methods

#### 2.2.1. NMR, IR Spectroscopy, and GPC Analysis

The ^1^H and ^13^C NMR spectra were recorded on JEOL 400 MHz and JEOL 100.5 MHz spectrometers (Tokyo, Japan) respectively; the solvent residual peak was used for referencing. The chemical shift values are on a *δ* scale, and the coupling constant values (*J*) are in Hertz. The HRMS data were collected on a Waters (Micromass) LCT1 using the ESI-TOF MS technique and Agilent 6210 ESI-TOF, Agilent Technologies, Santa Clara, CA, USA. Infrared spectra (IR) were recorded on a Perkin-Elmer FT-IR model 9 spectrometer. Molecular weight analysis ${\bar{M}}_{w}$, ${\bar{M}}_{n}$ and the Polydispersity Index (PDI) of polyethyleneglycol (PEG) unit containing amphiphiles was done using an Agilent GPC system equipped with an Agilent 1100 pump and a refractive index detector, using PLgel columns, with THF as an eluent at a flow rate of 1.0 mL min^−1^ using polystyrene standards.

#### 2.2.2. Critical Aggregation Concentration (CAC)

The critical aggregation concentration (CAC) value for the water-soluble synthesized amphiphiles was determined using the pendant drop method on a Tensionometer OCA 20 from Data Physics Instruments GmbH, Filderstadt, Germany. The samples at a concentration of 5 mg mL^−1^ in Milli-Q water were stirred vigorously for 20 h followed by two-fold serial dilution to form solutions of different concentrations. Subsequently, the surface tension values were recorded for these samples at 25 ± 5 °C until the deviation in the values stabilized. The CAC values were obtained from a plot of the surface tension values against the log [concentration of amphiphile].

#### 2.2.3. Dynamic Light Scattering (DLS) Measurement

DLS measurement was carried out using a Zetasizer Nano ZS analyzer (Malvern Instruments Ltd., Worcestershire, UK), integrated with 4 mW He–Ne laser, λ = 633 nm, using backscattering detection (scattering angle θ = 173°) equipped with an avalanche photodiode detector and thermostated sample chamber controlled by a thermoelectric Peltier. The respective amphiphiles were dissolved at a concentration of 5 mg mL^−1^ in Milli-Q water and vigorously stirred for at least 20 h at room temperature. The solutions were filtered via 0.45 μm polytetrafluoroethylene (PTFE) filter and allowed to stand undisturbed for 6 h at room temperature before measurements. Disposable UV-transparent cuvettes (12.5 × 12.5 × 45 mm, Sarstedt AG & Co, Nümbrecht, Germany) were used for all measurements with temperature maintained at 25 °C.

#### 2.2.4. Cryogenic Transmission Electron Microscopy (Cryo-TEM)

Morphological insights about the self-assembly formed by amphiphile **25** were obtained by recording the cryo-TEM image using a Tecnai F20 transmission electron microscope (FEI company, Hillsboro, OR, USA), operated at 160 kV accelerating voltage. For cryo sample preparation, droplets of the sample solution (5 mg of amphiphile in 1 mL of millipore water stirred, filtered) were applied to pre-hydrophilized perforated (1-µm hole diameter) carbon film-covered 200 mesh grids (R1/4 batch of the Quantifoil Micro Tools GmbH, Jena, Germany). The extra fluid was removed with a filter paper until an ultra-thin layer of the sample solution was obtained, spanning the holes of the carbon film. The samples were immediately vitrified by propelling the grids into liquid ethane at its freezing point (90 K) with a guillotine-like plunging device. Then, the vitrified samples were transferred to the microscope using a Gatan (Gatan, Inc., Pleasanton, CA, USA) cryoholder and stage (model 626). The samples were kept at a temperature of 94 K. Imaging was performed using the low-dose protocol of the microscope. Data were recorded by an Eagle 4k CCD-camera set to binning factor 2.

#### 2.2.5. Guest Encapsulation and Quantification

Nile red and dexamethasone encapsulation was done using thin film method and quantified by UV-visible spectra and high-performance liquid chromatography (HPLC) technique, respectively. Stock solutions of Nile red and dexamethasone were prepared in tetrahydrofuran and acetone respectively, and small volumes of stock (0.12 mg Nile red and 2.5 mg of dexamethasone, respectively per vial) were taken and allowed to evaporate to complete dryness under vacuum and darkness. Solutions of amphiphile in Millipore water each at a concentration of 5 mg mL^−1^ were added to the vial containing Nile red/dexamethasone and allowed to stir at 1200 rpm at room temperature for 20 h. Keeping in mind the aggregation of Nile red as observed by our group previously [29], we prepared a stock solution of Nile red in anhydrous tetrahydrofuran and added 40 µL from 1 × 10^−1^ M stock solution in vial to give 0.12 mg of Nile red in each vial. After stirring, the non-encapsulated fraction of guest was removed by filtering, slowly through a 0.45 μm PTFE filter. For the quantification of encapsulated Nile red, the encapsulated samples were lyophilized and re-dissolved in anhydrous methanol. The absorbance spectra were recorded on a Perkin-Elmer LAMBDA 950 UV/Vis/NIR spectrophotometer using standard disposable PMMA UV/Vis cuvettes with a path length of 1 cm from PLASTIBRAND. Fluorescence measurements were performed on a Jasco FP-6500 spectrofluorimeter using a variable slit system from 575 to 800 nm for Nile red. The fluorescence emission spectra were recorded by carrying out the excitation at 550 nm for Nile red, with the excitation and emission slit set at 5 nm. Transport capacity and efficiency were calculated using Lambert Beer law using a molar extinction coefficient of 45,000 M^−1^cm^−1^ at 552 nm for Nile red. The amount of dexamethasone encapsulated was quantified by lyophilizing the filtered sample and adding acetonitrile:water (2:3) and subjecting the solution for HPLC measurement using a dexamethasone calibration curve. To take into account the natural solubility of dexamethasone in water, a blank containing dexamethasone in water was prepared, analyzed by a similar method, and the value was subtracted from each amphiphile to get the effective loading efficiency. HPLC measurements were made on Knauer Smartline equipped with an RI detector 2300, UV detector 2550, and 1050 pump using RP Gemini C18 using an acetonitrile:water (40:60) solvent mixture as the mobile phase at a flow rate of 1 mL min^−1^ under an isocratic regime with a UV detector at 254 nm and 93 bar pressure. Microsoft Excel^®^ and Origin 2019 software were used for data analysis.

#### 2.2.6. Cytotoxicity Study

The cytotoxicity studies were performed using the 3-(4,5-dimethylthiazol-2-yl)-5-(3-carboxymethoxyphenyl)-2-(4-sulfophenyl)-2*H*-tetrazolium (MTS) assay. The studies for all amphiphiles were carried out in 1 X Phosphate-buffered saline (PBS buffer) at a series of concentrations of 2, 1, 0.5, and 0.1 mg mL^−1^ after 24 h using CellTiter 96^®^AQ_ueous_ One Solution Cell Proliferation Assay from Promega (Mannheim, Germany) as per the manufacturer’s instructions. A549 cells were seeded in a 96-well plate (4000 cells/well in 90 µL) and cultured overnight at 37 °C before adding the sample substances (10 µL) in serial dilutions (150, 100, 50, 25 μm). The surfactant, sodium dodecyl sulfate (SDS) (0.01%), and non-treated cells served as controls. For background subtraction, wells containing no cells but only samples were used. Cells were incubated for 24 h at 37 °C before the MTS solution (3-(4,5-dimethylthiazol-2-yl)-5-(3-carboxymethoxyphenyl)-2-(4-sulfophenyl)-2*H*-tetrazolium) (20 µL) was added. After an incubation period of 2 h and 30 min, absorbance was measured at a wavelength of 490 nm and a reference wavelength of 630 nm with a Tecan plate reader (Infinite pro200, TECAN-reader Tecan Group Ltd., Männedorf, Switzerland). Measurements were made in triplicate and repeated three times. Cell viability calculations were done by setting the non-treated control to 100% and the non-cell control to 0% by subtracting the background using Microsoft Excel. GraphPad Prism (5.01) served for data visualization.

### 2.3. Synthetic Procedures for Hydrophobic Backbone and Amphiphilic Architectures

#### 2.3.1. *D*-Glucitol Tris-acetonide (**2**)

*D*-Gluctiol tris-acetonide (**2**) was synthesized following the published procedure, and the spectral data obtained matched well with literature [29].

#### 2.3.2. *D*-Glucitol-3,4-Acetonide (**3**)

*D*-Glucitol-3,4-acetonide (**3**) was synthesized by following the procedure reported by Amber and co-workers with slight modifications [30]. Compound **2** (4.86 g) was dissolved in 70% acetic acid (100 mL) in a round-bottom flask followed by heating at 40 °C for 35 min. Then, the solvent was removed under vacuum at 40 °C, and the semi-solid residue thus obtained was taken up in boiling acetone (60 mL) and filtered to remove the solid residue formed. The filtrate was concentrated to around 20 mL and poured into boiling toluene (100 mL). This solution was concentrated under vacuum to remove the solvent to complete dryness. The solid residue was dried under a high vacuum, followed by washings with hexane (2 × 100 mL) and then recrystallized from aqueous acetone to give compound **3** in 45% yield. **^1^H NMR (400 MHz, DMSO-*d_6_*)** δ: 4.83–4.82 (m, 1H, OH), 4.54–4.51 (m, 1H, OH), 4.49–4.46 (m, 2H, OH), 3.99 (dd, *J* = 7.4, 1.7 Hz, 1H, H-3/H-4 ), 3.90 (t, *J* = 7.4 Hz, 1H, H-3/H-4), 3.55–3.48 (m, 2H, H-2/H-5, H1/H6), 3.45–3.36 (m, 3H, H-2/H-5, H1/H6), 3.31–3.30 (m, 1H), 1.29 (s, 3H, CH_3_), 1.27 (s, 3H, CH_3_). **^13^C NMR (100.5 MHz, DMSO-*d_6_*)** δ: 108.0 (Ca), 79.3, 75.8 (C-3, C-4), 73.6, 70.5 (C-2, C-5), 63.5, 63.3 (C-1, C-6), 27.4 (CH_3_), 26.9 (CH_3_).

#### 2.3.3. *D*-Glucitol-3,4-Diacetonide-5,6-Diazide (**5**)

The two primary hydroxyl groups of glucitol-3,4-monoacetonide were tosylated using a reported procedure with some modifications [31] to yield compound **4**, which was subsequently subjected to an azidation reaction to obtain glucitol-3,4-monoacetonide-1,6-diazide (**5**). Compound **3** (7 g) was added to 40 mL of pyridine under an inert atmosphere and was stirred for 10 min at −5 °C followed by the addition of *p*-toluenesulphonyl chloride (2.05 equivalent) in small portions, and the reaction mixture was allowed to stir for 3–4 h at −5 to 0 °C. The reaction mixture was poured into 100 mL of cold 6 N HCl solution and stirred to neutralize pyridine and then extracted with diethyl ether (3 × 150 mL). The organic layer was washed with saturated solution of sodium bicarbonate (2 × 150 mL). The combined organic layer was dried over anhydrous sodium sulfate followed by the removal of the solvent under reduced pressure and drying the residue to complete dryness to give compound **4**. Then, it was subjected to azidation by dissolving in 100 mL of Dimethylformamide (DMF) under N_2_ atmosphere and was charged with 5 equivalent of sodium azide followed by overnight heating at 90 °C. After completion of the reaction, as monitored by checking the TLC (ethyl acetate:petroleum ether::40:60), DMF was removed under reduced pressure, and the residue was extracted with ethyl acetate (3 × 100 mL). The combined organic layer was dried over anhydrous sodium sulfate. The crude residue so obtained was subjected to purification using column chromatography over silica gel using a gradient of ethyl acetate:petroleum ether as eluent to give compound **5** as a light yellow colored viscous oil in 53% yield. **^1^H NMR** (**400 MHz**, **CDCl_3_**): δ 4.03 (dd, *J* = 7.6, 3.0 Hz, 1H), 3.94 (s, 1H), 3.92–3.88 (m, 1H), 3.80–3.74 (m, 1H), 3.63 (dd, *J* = 12.5, 2.8 Hz, 1H), 3.49–3.39 (m, 3H), 2.81 (s, 2H, 2 × OH), 1.42 (s, 3H, CH_3_), 1.38 (s, 3H, CH_3_). **^13^C NMR** (**100.5 MHz**, **CDCl_3_**): δ 110.1 (Ca), 80.2, 76.5 (C-3, C-4), 72.3, 69.6 (C-2, C-5), 54.5, 54.0 (C-1, C-6), 27.1 (CH_3_), 27.0 (CH_3_). **HRMS** (*m/z*):[M+ Na]^+^ calculated for C_9_H_16_N_6_O_4_; 295.1125, found 295.1166. **IR** (**DCM**) **ν_max_** (**cm^−1^**): 3416, 2989, 2932, 2102, 1445, 1377, 1259, 1164, 1121, 1071.

#### 2.3.4. Compound **6**

To a solution of compound **5** (1.83 mmol) and phenylacetylene (4.593 mmol) in THF/H_2_O mixture (3:1, 20 mL), sodium ascorbate (1.470 mmol) and CuSO_4_.5H_2_O (0.735 mmol) were added. The reaction mixture was stirred at 30 °C for 24 h. After monitoring the completion of reaction on TLC, THF was removed under vacuum. The residue was extracted with chloroform, the organic layer was washed with saturated solution of Ethylenediamine tetraacetic acid (EDTA) (3 × 100 mL), and the organic layer was dried over anhydrous sodium sulfate. The collected organic layer was concentrated under vacuo. The crude product was subjected to purification by column chromatography on silica using a gradient of methanol: chloroform as an eluent to yield compound **6** as an off-white solid in 75% yield. **^1^H NMR** (**400 MHz**, **CDCl_3_**): δ 7.84 (s, 1H, H-5′), 7.82 (s, 1H, H-5a′), 7.55 (dd, *J* = 6.9, 1.3 Hz, 2H, ArH), 7.48 (dd, *J* = 4.9, 2.2 Hz, 2H, ArH), 7.28–7.16 (m, 6H, ArH), 5.64 (brs, 1H, OH), 4.95 (brs, 1H, OH), 4.76 (d, *J* = 14.1 Hz, 1H, H-1/H-6), 4.68 (d, *J* = 13.6 Hz, 1H, H-1/H-6), 4.48–4.30 (m, 3H, H-2/H-5, H-1/H-6), 4.17 (d, *J* = 6.9 Hz, 1H, H-3/H-4), 4.07 (dd, *J* = 19.5, 9.0 Hz, 2H, H-2/H-5 & H-3/H-4), 1.47 (s, 3H, CH_3_), 1.41 (s, 3H, CH_3_). **^13^C NMR** (**100.5 MHz**, **CDCl_3_**): δ147.5, 147.3 (C-4′, C-4a′), 130.0, 129.9 (C-1″, C-1a″), 128.9 (C-3″, C-5″, C-3a″, C-5a″), 128.3 (ArC), 128.2 (ArC), 125.6 (ArC), 125.5 (ArC), 121.8, 121.6 (C-5′, C-5a′), 110.4 (Ca), 81.0 (C-3/C-4), 72.4, 69.3 (C-2, C-5), 54.1, 53.8 (C-1, C-6), 27.2 (CH_3_), 27.0 (CH_3_). **HRMS** (*m/z*):[M+ H]^+^ calculated for C_25_H_28_N_6_O_4_ 477.2245; found 477.2245. **IR** (**KBr**) **ν_max_**(**cm^−1^**): 3374, 3142, 2988, 2931, 1610, 1484, 1466, 1441, 1373, 1227, 1164, 1113, 1075.

#### 2.3.5. 1-(Prop-2-yn-1-yloxy)naphthalene **9**

Compound **9** was synthesized following a literature report with some modifications [32]. Briefly, naphthalen-1-ol (**8**) (3.6 g, 0.025 mol) was dissolved in anhydrous acetone (50 mL) under nitrogen atmosphere followed by the addition of K_2_CO_3_ (5.17 g, 0.037 g), and the reaction mixture was then stirred for 30 min. Then, propargyl bromide (3.57 g, 0.299 mol) was added slowly, and the reaction mixture was refluxed at 50 °C for 12 h. On completion of the reaction, K_2_CO_3_ was filtered off, and the solvent was collected and concentrated under vacuo followed by extraction with ethyl acetate (2 × 100 mL). The organic layer was collected, dried over anhydrous sodium sulfate, and concentrated under vacuo to complete dryness. The crude residue was washed with hexane (3 × 100 mL) under sonication and dried under vacuum to give compound **9** in 90% yield.

#### 2.3.6. Compound **10**

To a solution of compound **5** (500 mg, 1.83 mmol) and 1-(prop-2-yn-1-yloxy)naphthalene (**9**) (832.97 mg, 4.575 mmol) in Dichloromethane (DCM), tris(triphenylphosphine) copper(I) bromide (34 mg, 0.036 mmol) and *N*,*N*-diisopropylethylamine (DIPEA) (2.5 mL, 11.41 mmol) were added. The reaction mixture was allowed to stir for 24 h at 30 °C. The progress of reaction was monitored using TLC (methanol:chloroform: 5:95) and on completion of the reaction, DCM was evaporated under vacuum. The residue was extracted with chloroform (3 × 100 mL). The collected organic layer was concentrated under vacuum. The crude product was subjected to purification by column chromatography on silica gel using a gradient of methanol:chloroform as an eluent to yield compound **10** as an off-white solid in 78% yield.

**^1^H NMR** (**400 MHz, CDCl_3_**): δ 8.19 (s, 1H, ArH), 8.17 (s, 1H, ArH), 7.78–7.75 (m, 4H, H-5′, H-5a′, 2 × ArH), 7.46–7.38 (m, 6H, ArH), 7.31–7.27 (m, 2H, ArH), 6.85–6.81 (m, 2H, ArH), 5.27–5.20 (m, 4H, H1a″/H1″), 4.92 (s, brs, 1H, OH), 4.74 (dd, *J* = 14.1, 2.1 Hz, 1H, H-1a/H-1b/H-6a/H-6b), 4.62 (dd, *J* = 14.1, 3.1 Hz, 1H, H-1/H-6), 4.46–4.36 (m, 2H, H-1/H-6), 4.28 (brs, 1H), 4.11–4.02 (m, 2H, H-2/H-5, H-3/H-4), 3.93–3.92 (m, 1H, H-3/H-4), 3.90–3.85 (m, 1H), 1.42 (s, 3H, CH_3_), 1.38 (s, 3H, CH_3_). **^13^C NMR** (**100.5 MHz**, **CDCl_3_**): δ 153.8 (ArC), 144.2, 144.0 (C-4′, C-4a′), 134.5 (ArC), 127.6 (ArC), 126.6 (ArC), 125.8 (ArC), 125.5 (ArC), 125.4 (ArC), 124.6, 124.5 (C-5′/C-5a′), 122.0 (ArC), 120.9 (ArC), 110.3 (Ca), 105.3 (ArC), 81.0, 76.7 (C-3, C-4), 72.3, 69.3 (C-2, C-5), 62.1 (C-1″, C-1a″), 54.1, 53.5 (C-1, C-6), 27.1 (CH_3_), 26.9 (CH_3_). **HRMS** (*m/z*):[M+ H]^+^ calculated for C_35_H_36_N_6_O_6_; 637.2769, found 637.2800. **IR** (**KBr**) **ν_max_**(**cm^−1^**): 3394, 2986, 2927, 1596, 1580, 1508, 1460, 1438, 1395, 1269, 1239, 1158, 1098, 1069, 1019.

### 2.4. General Procedure for Synthesis of *Compounds **7** and **11***

To a solution of compound **6** (500 mg, 1.049 mmol) in anhydrous THF (150 mL) under nitrogen atmosphere, sodium hydride (4.197 mmol, 167 mg) was added in small portions over 30 min, and the reaction mixture was allowed to stir for the additional 1 h post addition of sodium hydride. Propargyl bromide (375 mg, 3.147 mmol) was added slowly to the reaction mixture and allowed to stir at 30 °C for 20 h. On completion of the reaction, which was monitored using TLC (ethyl acetate:petroleum ether:50:50), THF was removed under vacuum followed by quenching of the reaction mixture with water and extraction with ethyl acetate (3 × 100 mL). The combined organic layer was washed with brine solution (2 × 100 mL) and dried over anhydrous sodium sulfate. The collected organic layer was concentrated under vacuo. A similar procedure was followed for the synthesis of compound **11**. The crude product was subjected to purification by column chromatography on silica gel using a gradient of methanol:chloroform as an eluent to yield pure compound (**7/11**). Compound **7** was obtained as an off-white fluffy hygroscopic solid in 55% yield. **^1^H NMR** (**400 MHz**, **CDCl_3_**): δ 7.89 (s, 2H, H-5′, H-5a′), 7.84–7.81 (m, 4H, ArH), 7.42 (t, *J* = 7.7 Hz, 4H, ArH), 7.35–7.31 (m, 2H, ArH), 4.84 (dd, *J* = 14.6, 2.8 Hz, 1H, H-1/H-6), 4.64 (dd, *J* = 14.1, 5.1 Hz, 1H, H-1/H-6), 4.59–4.51 (m, 2H, H-1/H-6), 4.21 (dd, *J =* 16.3, 2.4 Hz, 1H, H1‴/H1a‴), 4.13–3.93 (7H, m, H-2, H-3, H-4, H-5, H-1‴, H-1a‴), 2.39 (t, *J* = 2.4 Hz, 1H), 2.18 (t, *J* = 2.4 Hz, 1H, H-3‴, H-3a‴), 1.49 (s, 3H, CH_3_), 1.47 (s, 3H, CH_3_). **^13^C NMR** (**100.5 MHz**, **CDCl_3_**): δ147.9, 147.7 (C-4′, C-4a′), 130.6, 130.5, 128.9, 128.3, 128.2, 125.8, 125.7, 121.5, 121.2 (C-5′, C-5a′), 110.4 (Ca), 80.1 (C-3/C-4), 78.6, 78.5 (C-2‴, C-2a‴), 78.0 (C-3/C-4), 77.0 (C-2/C-5), 76.3, 75.5 (C-3‴, C-3a‴), 74.7 (C-2/C-5), 59.2, 57.6 (C-1‴, C-1a‴), 51.5, 50.4 (C-1, C-6), 27.2 (CH_3_), 26.7 (CH_3_). **HRMS** (*m/z*):[M+ H]^+^ calculated for C_31_H_32_N_6_O_4;_553.2558, found 553.2560. **IR** (**KBr**) **ν_max_** (**cm^−1^**): 3448, 3293, 3138, 2988, 2925, 2857, 2373, 2118 (C≡C), 1618, 1466, 1442, 1374, 1227, 1166, 1126, 1081.

Compound **11** was obtained as a white fluffy hygroscopic solid in 63% yield. **^1^H NMR** (**400 MHz**, **CDCl_3_**): δ 8.23 (d, *J* = 7.9 Hz, 2H, ArH), 7.84–7.75 (m, 4H, 2 × ArH, H-5, H-5a′), 7.50–7.42 (m, 6H, ArH), 7.36 (t, *J* = 8.0 Hz, 2H, ArH), 6.95 (d, *J* = 7.5 Hz, 2H, ArH), 5.43 (s, 2H, H-1″/H-1a″), 5.41 (s, 2H, H-1″/H-1a″), 4.81 (dd, *J* = 14.6, 2.8 Hz, 1H, H-1/H-6), 4.60 (dd, *J* = 13.9, 5.2 Hz, 1H, H-1/H-6), 4.55–4.44 (m, 2H, H-1/H-6), 4.11 (dd, *J* = 16.1, 2.3 Hz, 1H, H-1′′′′/H-1a′′′′), 4.07–3.83 (m, 7H, H-1′′′′, H-1a′′′′, H-2, H-5, H-3, H-4), 2.40 (t, *J* = 2.3 Hz, 1H), 2.22 (t, *J* = 2.2 Hz, 1H, H-3′′′′, H-3a′′′′), 1.40 (s, 3H, CH_3_), 1.38 (s, 3H, CH_3_). **^13^C NMR** (**100.5 MHz**, **CDCl_3_**): δ 153.9 (ArC), 144.6, 144.3 (C-4′, C-4a′), 134.6 (ArC), 127.6 (ArC), 126.6 (ArC), 125.8 (ArC), 125.7 (ArC), 125.4 (ArC), 124.5, 124.3 (C-5′, C-5a′), 122.0 (ArC), 121.0 (ArC), 110.5 (Ca), 105.4 (ArC), 80.1 (C-3/C-4), 78.5, 78.5 (C-2′′′′, C-2a′′′′), 77.9 (C-3/C-4), 76.7, 76.4 (C-3′′′′, C-3a′′′′), 75.6, 74.6(C-2, C-5), 62.4, 62.4 (C-1″, C-1a″), 59.1, 57.5 (C-1′′′′, C-1a′′′′), 51.5, 50.4 (C-1, C-6), 27.1 (CH_3_), 26.6 (CH_3_). **HRMS** (*m/z*):[M+ H]^+^ calculated for C_41_H_40_N_6_O_6_ 713.3082; found 713.3121. **IR** (**KBr**) **ν_max_**(**cm^−1^**): 3436, 3286, 3145, 3054, 2987, 2926, 2118 (C≡C), 1618, 1596, 1580, 1508, 1460, 1394, 1350, 1269, 1239, 1177, 1157, 1097, 1050, 1019.

### 2.5. General Procedure for the Synthesis of Amphiphiles *(**20**–**25**)*

Compound **7/11** (1 equivalent) and corresponding azide i.e., methoxypoly[ethylene glycol] azide (*M_w_* = 550, 1100 g/mol) or glycerol azide, triglycerol azide (3 equivalent) were taken in a 100 mL flask and dissolved in anhydrous dichloromethane (30 mL) for reaction with mPEG azide and in DCM/DMF (1:1) mixture (30 mL) for reaction with glycerol azide/triglycerol azide under nitrogen atmosphere. Tris(triphenylphosphine)copper(I) bromide (0.05 equivalent) and *N*,*N*-diisopropylethylamine (DIPEA) (12.5 equivalent) were added to the reaction mixture and allowed to stir for 72 h at 30 °C. Progress of the reaction was monitored using TLC (methanol:chloroform: 2:98/10:90) by the disappearance of the hydrophobic core (**7/11**). On completion of reaction, the solvent was evaporated completely followed by washing with hexane using the sonication of the residual crude product to remove excess/unreacted reactants and copper catalyst tris(triphenylphosphine)copper(I) bromide. The crude residue was subjected to purification using column chromatography/dialysis (2000 MWCO dialysis tubing; chloroform; 48 h) to yield the purified compounds (**20–25**) in 35–55% yield.

#### 2.5.1. Synthesis of Amphiphile **20**

Amphiphile **20** was synthesized by the reaction of compound **7** and glyceryl azide (**14**) in DCM/DMF (1:1) following the general procedure to yield compound **20** as a light yellow hygroscopic solid in 52% yield. **^1^H NMR** (**400 MHz**, **DMSO-*d_6_***): δ8.63 and 8.59 (2 x s, 2 × 1H, H-5′′′′, H-5a′′′′), 8.04 (s, 1H, H-5/H-5′), 7.88–7.85 (m, 4H, ArH), 7.79 (s, 1H, H-5/H-5′), 7.47–7.43 (m, 4H, ArH), 7.35–7.32 (m, 2H, ArH), 5.06–5.01 (m, 4H, 4 × OH), 4.80–4.76 (m, 1H, H-1/H-6), 4.60–4.43 (m, 7H, 3 × H1/H6, 2 × H1‴, H1a‴), 4.36–4.33 (m, 1H, Hb/Hb′), 4.17–4.07 (m, 5H, Hb/Hb′, H-2, H-3, H-4, H-5), 3.77–3.72 (m 8H, Hc, Hc′), 1.40 (s, 3H, CH_3_), 1.37 (s, 3H, CH_3_). **^13^C NMR** (**100.5 MHz**, **DMSO-*d_6_***): δ 146.4, 146.3 (C-4′, C-4a′), 142.8, 142.7 (C-4′′′′, C-4a′′′′), 130.7 (ArC), 130.7 (ArC), 128.9 (ArC), 127.8 (ArC), 125.2 (ArC), 123.5, 123.0 (C5′, C-5a′), 122.6, 122.3 (C-5′′′′, C-5a′′′′), 109.3 (Ca), 79.1, 78.1 (C-3, C-4), 77.0, 75.1 (C-2, C-5), 64.9, 64.7 (C-1‴, C-1a‴), 64.6, 63.3 (C-b, C-b′), 60.5 (C-c, C-c′), 51.5, 50.5 (C-1, C-6), 26.9 (CH_3_), 26.7 (CH_3_). **HRMS** (*m/z*):[M+ H]^+^ calculated for C_37_H_46_N_12_O_8_ 787.3634; found 787.3635. **IR** (**KBr**) **ν_max_**(**cm^−1^**): 3400, 2925, 1637, 1382, 1228, 1080, 767, 696.

#### 2.5.2. Synthesis of Amphiphile **21**

Amphiphile **21** was synthesized by reacting compound **7** with methoxypoly[ethylene glycol] azide (M_w_ = 550 g/mol) (**18**) in DCM following the general procedure to yield compound **21** as a brown-colored low melting solid in 50% yield. **^1^H NMR** (**400 MHz**, **CDCl_3_**): δ8.01 and 7.99 (2 × s, 2 × H, H-5′′′′, H-5a′′′′), 7.84–7.81 (m, 4H, ArH), 7.69 (s, 1H, H-5′/H-5a′), 7.40 (t, *J* = 7.4 Hz, 4H, ArH), 7.32–7.28 (m, 3H, 2 ×ArH, H-5′/H5a′), 4.81 (d, *J* = 14.2 Hz, 1H, H-1/H-6), 4.56 (dd, *J* = 12.9, 7.6 Hz, 2H, H-1/H-6), 4.50–4.44 (m, 4H), 4.36 (d, *J* = 12.2 Hz, 1H), 4.28 (d, *J* = 12.2 Hz, 1H), 4.24–4.17 (m, 2H), 4.13–4.01 (m, 2H), 3.95 (t, *J* = 7.8 Hz, 1H), 3.83–3.79 (m, 3H), 3.69 (t, *J* = 5.2 Hz, 2H), 3.63–3.50 (m, 97H, OCH_2_CH_2_), 3.36 (s, 6H, 2 x OCH_3_), 1.41 (s, 3H, CH_3_), 1.40 (s, 3H, CH_3_). **^13^C NMR** (**100.5 MHz**, **CDCl_3_**): δ147.7, 147.5 (C-4′, C-4a′), 143.9, 143.4 (C-4′′′′, C-4a′′′′), 130.7 (ArC), 130.6 (ArC), 129.0 (ArC), 128.9 (ArC), 128.2 (ArC), 128.1 (ArC), 125.7 (ArC), 124.6, 124.0 (C-5′′′′, C-5a′′′′), 121.5, 121.5 (C-5′, C-5a′), 110.1 (Ca), 80.4, 78.6 (C-3, C-4), 74.7 (C-2, C-5), 72.0 (C-e), 70.6 (–OCH_2_CH_2_), 69.2 (C-c), 64.7, 63.3 (C-1‴, C-1a‴), 59.1 (OMe), 51.9, 51.1, 50.3, 49.9 (C-1, C-6, C-b), 27.1 (CH_3_), 26.7 (CH_3_). **GPC (THF, 1 mL min^−1^)**: ${\bar{M}}_{w}$ = 1651 g mol^−1^, ${\bar{M}}_{n}$ = 1508 g mol^−1^, ${\bar{M}}_{z}\;$= 1769 g mol^−1^, PDI = 1.09. **IR** (**neat**) **ν_max_**(**cm^−1^**): 3572, 3131, 2867, 1463, 1349, 1248, 1089.

#### 2.5.3. Synthesis of Amphiphile **22**

Amphiphile **22** was synthesized by reacting compound **11** with methoxypoly[ethylene glycol]azide M_w_ = 1100 g/mol (**19**) in DCM following the general procedure to yield compound **22** as a brown semi-solid in 55% yield. **^1^H NMR** (**400 MHz**, **CDCl_3_**): δ8.01 (s, 1H) and 8.00 (2 × s, 2 × 1H, H-5′′′′, H-5a′′′′), 7.85–7.82 (m, 4H, ArH), 7.70 (s, 1H, H-5′/H-5a′), 7.41 (t, *J* = 7.5 Hz, 4H), 7.33–7.28 (m, 3H, 2 × ArH, H-5′/H-5a′), 4.81 (dd, *J* = 14.4, 2.7 Hz, 1H), 4.58–4.54 (m, 2H), 4.50–4.46 (m, 4H), 4.43–4.29 (m, 3H), 4.26–4.20 (m, 2H), 4.10–4.02 (m, 2H), 3.94 (t, *J* = 7.9 Hz, 1H), 3.84–3.77 (m, 4H), 3.70–3.67 (m, 4H), 3.63–3.54 (m, 190H, OCH_2_CH_2_), 3.36 (s, 6H, 2 × OCH_3_), 1.42 (s, 3H, CH_3_), 1.40 (s, 3H, CH_3_). **^13^C NMR** (**100.5 MHz**, **CDCl_3_**): δ147.6, 147.5 (C-4′, C-4a′), 143.5, 143.3 (C-4′′′′, C-4a′′′′), 130.6 (ArC), 130.6 (ArC), 129.0 (ArC), 128.9 (ArC), 128.2 (ArC), 128.1 (ArC), 125.7 (ArC), 124.6, 124.0 (C-5′′′′, C-5a′′′′), 121.5, 121.5 (C-5′, C-5a′), 110.1 (C-a), 80.4, 78.7 (C-3, C-4), 74.6 (C-2, C-5), 72.0 (C-e, C-e′), 70.6 (–CH_2_-CH_2_-O), 69.2 (C-c), 64.7, 63.3 (C-1‴, C-1a‴), 59.1 (OMe), 51.9, 51.1, 50.3, 49.9 (C-1, C-6, C-b), 27.0 (CH_3_), 26.7 (CH_3_). **GPC (THF, 1 mL min^−1^)**: ${\bar{M}}_{w}$ = 2861 g mol^−1^, ${\bar{M}}_{n}$ = 2722 g mol^−1^, ${\bar{M}}_{z}$ = 2995 g mol^−1^, PDI = 1.05. **IR** (**neat**)**ν_max_**(**cm^−1^**): 3526, 2867, 1643, 1455, 1348, 1298, 1249, 1088.

#### 2.5.4. Synthesis of Amphiphile **23**

Amphiphile **23** was synthesized by reacting compound **7** with triglycerol azide (**17**) in DCM:DMF (1:1) following the general procedure to yield compound **23** as an off-white hygroscopic solid in 40% yield. **^1^H NMR** (**400 MHz**, **Methanol-*d_3_***): δ 8.40–8.28 (m, 2H, H-5′′′′, H-5a′′′′), 8.14–7.97 (m, 1H, H-5′/H-5a′), 7.84 (t, *J* = 7.9 Hz, 4H, ArH), 7.73–7.50 (m, 1H, H-5′/H-5a′), 7.47–7.42 (m, 4H, ArH), 7.37–7.35 (m, 2H, ArH), 4.99 (dd, *J* = 10.5, 5.0 Hz, 1H, H-b/H-b′), 4.87–4.29 (m, 11H, H-1a, H-1b, H-6a, H-6b, H-b/H-b′, 6 × OH), 4.28–4.07 (m, 4H, H-2, H-3, H-4, H-5), 4.05–3.96 (m, 2H, 2 × OH), 3.96–3.85 (m, 4H, H-1‴, H-1a‴), 3.82–3.36 (m, 28H, Dendron), 1.44 (s, 3H, CH_3_), 1.42 (s, 3H, CH_3_). **^13^C NMR** (**100.5 MHz**, **Methanol-*d_3_***): δ148.7, 148.7 (C-4′, C-4a′), 144.7 (C-4′′′′,C-4a′′′′), 131.6 (ArC), 131.5 (ArC), 130.1 (ArC), 130.0 (ArC), 129.4 (ArC), 129.4 (ArC), 126.8 (ArC), 126.8 (ArC), 125.5 (C5′, C5a′), 124.9, 123.9 (C-5′′′′, C-5a′′′′), 123.6 (ArC), 111.2 (Ca), 81.6, 80.1 (C-3, C-4), 78.5, 76.1 (C-2, C-5), 73.9, 73.7, 73.7, 72.2 (C-c), 72.1, 72.0, 72.0, 71.9, 71.1 (C-e), 71.0 (C-d), 65.5 (C-1‴, C-1a‴), 64.2 (C-f), 62.3, 62.0 (C-b), 53.1, 51.9 (C-1, C-6), 27.3 (CH_3_), 26.9 (CH_3_). **HRMS** (*m/z*):[M+ H]^+^ calculated for C_49_H_70_N_12_O_16_ 1083.5106, found 1083.5111. **IR** (**neat**) **ν_max_**(**cm^−1^**): 3308, 2943, 2831, 2519, 2341, 2227, 2044, 1448, 1419, 1116, 1027, 1020.

#### 2.5.5. Synthesis of Amphiphile **24**

Amphiphile **24** was synthesized by reacting compound **11** with methoxypoly[ethylene glycol]azide M_w_ = 1100 g/mol (**19**) in DCM/DMF (1:1) by following the general procedure to yield compound **24** as a light brown-colored low melting solid in 38% yield. **^1^H NMR** (**400 MHz**, **CDCl_3_**): δ8.18 (d, *J* = 8.1 Hz, 2H, ArH), 7.91 (s, 1H), 7.88 (s, 1H), 7.77 (s, 1H), 7.75 (s, 1H), 7.61 (s, 1H), 7.49–7.29 (m, 9H, ArH), 6.97 (t, *J* = 7.5 Hz, 2H, ArH), 5.45–5.36 (m, 4H, H-1″, H1a″), 4.80 (dd, *J* = 14.3, 2.2 Hz, 1H, H-1a/H-1b/H-6a/H-6b), 4.54–4.20 (m, 11H), 4.14 (d, *J* = 12.0 Hz, 1H), 3.99–3.85 (m, 3H), 3.79–3.75 (m, 2H), 3.64–3.52 (m, 194H, OCH_2_CH_2_), 3.37 (s, 6H, 2 × OCH_3_), 1.34 (s, 3H, CH_3_), 1.32 (s, 3H, CH_3_). **^13^C NMR** (**100.5 MHz**, **CDCl_3_**): δ153.9 (ArC), 144.3, 144.1 (C-4′,C-4a′), 143.5, 143.2 (C-4′′′′,C-4a′′′′), 134.5 (ArC), 127.5 (ArC), 126.5 (ArC), 125.9 (ArC), 125.6 (ArC), 125.6 (ArC), 125.4 (ArC), 124.6, 124.1, 122.0, 122.0 (C-5′, C-5a′, C-5′′′′, C-5a′′′′), 120.8 (ArC), 110.1 (C-a), 105.4 (ArC), 80.3, 78.9 (C-3, C-4), 76.4, 74.4 (C-2, C-5), 72.0 (C-e, C-e’), 70.6 (O-CH_2_-CH_2_), 69.3 (C-c), 64.7, 63.2 (C-1″, C-1a″), 62.3 (C-1′′′′, C-1a′′′′), 59.1 (OMe), 51.8, 51.0, 50.2, 49.99 (C-1, C-6, C-b), 27.07 (CH_3_), 26.64 (CH_3_). GPC (THF, 1 mL min^−1^): ${\bar{M}}_{w}$ = 2278 g mol^−1^, ${\bar{M}}_{n}$ = 1800 g mol^−1^, ${\bar{M}}_{z}$ = 2606 g mol^−1^, PDI = 1.26. **IR** (**neat**) **ν_max_**(**cm^−1^**): 3503, 2867, 1653, 1579, 1461, 1348, 1298, 1268, 1244, 1091.

#### 2.5.6. Synthesis of Amphiphile **25**

Amphiphile **25** was synthesized by reacting compound **11** with triglycerol azide (17) in DCM/DMF (1:1) by following the general procedure as a light orange hygroscopic solid in 36% yield. **^1^H NMR** (**400 MHz**,**Methanol-*d*_3_**): δ 8.21 (t, *J* = 4.5 Hz, 2H, ArH), 8.16–7.94 (m, 3H, CH triazole), 7.88–7.73 (m, 3H, 2 × ArH, 1 × CH triazole), 7.55–7.32 (m, 8H, ArH), 7.08–7.05 (m, 2H, ArH), 5.38 (s, 4H, H-1′′′′, H-1a′′′′), 5.01–4.93 (m, 1H, H-b), 4.87–4.78 (m, 2H, H-b, H-1a/H-1b/H-6a/H-6b), 4.66–4.44 (m, *J* = 20.4, 14.8 Hz, 5H, 3 × H1a/H1b/H6a/H6b, 2 × H1a′′′′/H1a′′′′), 4.44–4.32 (m, 1H, 1 × H1a′′′′/H1a′′′′), 4.24–3.33 (m, 41H, dendron, H-2, H-3, H-4, H-5, H-1a/H-6a, 1 × H1a′′′′/H1a′′′′), 1.34 (s, 3H, CH_3_), 1.31 (s, 3H, CH_3_). **^13^C NMR** (**100.5 MHz**,**Methanol-*d*_3_**): δ 155.1 (ArC), 145.1, 144.5 (C4′, C4a′, C4′′′′, C4a′′′′), 136.0 (ArC), 135.9 (ArC), 128.5 (ArC), 127.5 (ArC), 127.1 (ArC), 126.9 (ArC), 126.9 (ArC), 126.8 (ArC), 126.3 (ArC), 125.5, 125.1,122.9, 122.8 (C-5′, C-5a′, C-5′′′′, C-5a′′′′), 121.8 (ArC), 111.2 (Ca), 106.7 (ArC), 106.6 (ArC), 81.5, 80.1 (C-3, C-4), 78.2, 75.7 (C-2, C-5), 73.8, 73.7, 73.6 (C-c), 72.6, 72.0, 71.9 (C-e), 71.0 (C-d), 65.5 (C-1‴/C-1a‴), 64.2 (C-f), 64.0, 62.7 (C-1′′′′, C-1a′′′′), 62.2, 62.0 (C-b), 52.8, 51.6 (C-1, C-6), 27.3 (CH_3_), 26.8 (CH_3_). **HRMS** (*m/z*):[M+ H]^+^calculated for C_59_H_78_N_12_O_18_ 1243.5630, found 1243.5624. **IR** (**neat**) **ν_max_**(**cm^−1^**): 3322, 2943, 2921, 2831, 2514, 2226, 2044, 1658, 1448, 1415, 1117, 1010.

## 3. Results and Discussion

**Figure 1:**
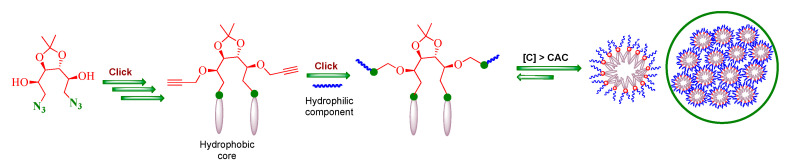
Proposed schematic representation of self-assembly formed by amphiphilic architectures in aqueous solution.

In an endeavor to create novel cytocompatible amphiphilic constructs as nanocarriers for biomedical applications, we have synthesized a series of *D*-glucitol-based amphiphiles employing biocompatible and green hydrophilic moieties and aromatic hydrophobic moieties. The modified glucitol core and hydrophobic backbone were synthesized and then coupled with hydrophilic components using copper-catalyzed “1,3-dipolar cycloaddition reaction” to synthesize the amphiphilic constructs, as depicted in Scheme 1. Their self-assembling tendency as illustrated in Figure 1 in aqueous medium was studied using dynamic light scattering (DLS), surface tension measurements, and cryogenic electron transmission microscopy (cryo-TEM). Subsequently, the cytotoxicity profile and ability of the self-assembled aggregates to encapsulate hydrophobic guests was evaluated.

### 3.1. Synthesis and Characterization

Glucitol-3,4-acetonide-1,6-diazide (**5**) was synthesized from *D*-glucitol in four steps as outlined in Scheme 1.

*D*-Glucitol was first converted to its tris-acetonide (**2**) using acetone and catalytic amount of conc. H_2_SO_4_ followed by its conversion to glucitol-3,4-monoacetonide (**3**) using 70% acetic acid (Figure S1, Supplementary Materials (SM)) [29]. The two primary hydroxyl groups of glucitol-3,4-monoacetonide were tosylated to yield compound **4**, which was subsequently subjected to an azidation reaction to obtain glucitol-3,4-monoacetonide-1,6-diazide (**5**). The structure of compound **5** was confirmed on the basis of its spectral data (Figure S2). In the IR spectrum of compound **5**, a signal around 2100 cm^−1^ confirmed the presence of an azide group. In the ^1^H NMR spectrum (Figure S2), two singlets integrating for three protons each were recorded at δ 1.42 and 1.38 ppm and were assigned to the two methyl groups of monoacetonide moiety. The methylene carbons in the diazide showed characteristic upfield shift as compared to the precursor compound **3** from δ 63.52 ppm and 63.30 ppm (Figure S1) to δ 54.55 ppm and 54.03 ppm on azidation at the primary hydroxyl group of the glucitol-3,4-monoacetonide (Figure S3). The methyl, methylene, and methine carbons in compound **5** were identified on the basis of Distortionless enhancement by polarization transfer (DEPT) data (Figure S3). Then, *D*-glucitol-3,4-monoacetonide-1,6-diazide (**5**) was grafted with phenyl acetylene/1-(prop-2-yn-1-yloxy)naphthalene using the ‘click chemistry’ approach to yield compounds **6** and **10** (Figures S4–S6). The coupling reaction was monitored by the disappearance of the peak for azide at around 2100 cm^−1^ in the IR spectrum. The structure of compound **6** was further established on the basis of appearance of peaks in the aromatic region in the ^1^H NMR spectrum (Figure S4). The formation of the triazole rings was also confirmed by the appearance of two singlets corresponding to H-5′/H-5a′ each integrating for one proton in the ^1^H NMR spectrum at around δ 7.8 ppm, while the corresponding carbons i.e., C-5′/C-5a′ appeared at about 121 ppm in the ^13^C NMR spectrum (Figures S4 and S5). The quaternary carbon (C-4′/C-4′a) of the triazole ring was observed at about δ 147 ppm assigned on the basis of the ^2^D HETCOR spectrum (Figure S5). The propargylation of compound **6**/**10** was carried out using sodium hydride and propargyl bromide to yield compounds **7/11** (Scheme 1). The successful conversion of compound **6/10** to its propargylated derivative **7/11** was confirmed from the IR spectrum, which showed a signal at around 3200 cm^−1^ (C-H) and 2100 cm^−1^ (C≡C). In addition, the structural confirmation of the final products was done by ^1^H and ^13^C NMR spectrum (Figures S7–S9).

**Scheme 2:**
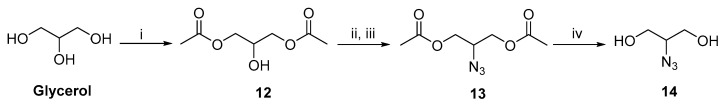
Synthesis of glyceryl azide. (i) Novozym-435, vinyl acetate, THF, 37 °C; (ii) mesyl chloride, triethylamine, DCM, 0–25 °C, 2h; (iii) NaN_3_, DMF, reflux, 12 h; (**iv**) K_2_CO_3_, EtOH, 12 h.

**Scheme 3:**
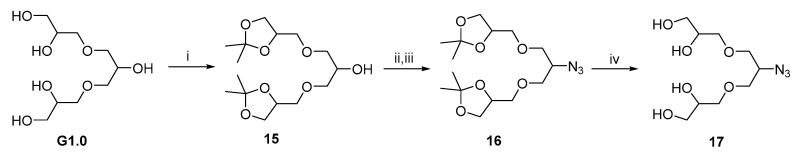
Synthesis of triglyceryl azide. (i) 2,2-dimethoxypropane, pTSA, 12 h; (ii) mesyl chloride, triethylamine, DCM, 0–25 °C, 2h; (iii) NaN3, DMF, reflux, 12 h; (**iv**) Dowex-50 resin, MeOH, 50 °C, 12 h.

**Scheme 4:**
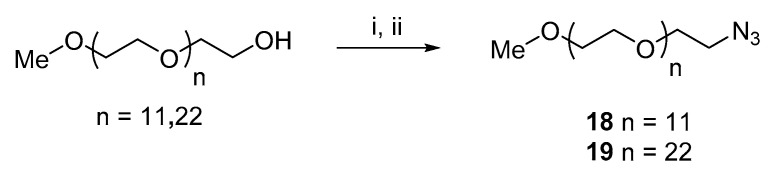
Synthesis of methoxy polyethyleneglycol (PEG) azide. (i) mesyl chloride, triethylamine, THF, 0–25 °C, 5h; (ii) NaN_3_, DMF, 30 °C, 48 h.

Glyceryl azide (**14**) was synthesized from commercially available glycerol in four steps by following a chemoenzymatic approach, as shown in Scheme 2. Firstly, glycerol was subjected to biocatalytic acylation using an immobilized enzyme *Candida antarctica* lipase (Novozym-435) by following the literature procedure to yield 2-hydroxypropane-1,3-diyl diacetate (**12**) (see Supplementary Materials) [33]. Then, the secondary hydroxyl group of compound **12** was subjected to mesylation followed by an azidation reaction to yield compound **13**. The deacetylation of compound **13** using K_2_CO_3_ led us to obtain the monomer 2-azido-1,3-propanediol (14). Scheme 3 depicts the steps followed for the synthesis of triglyceryl azide (**17**), and Scheme 4 outlines the synthetic strategy followed for monomethoxypolyethylene glycol azides (**18, 19**) synthesized from the available precursors, triglycerol (G1.0) and monomethoxypolyethylene glycol, respectively, following the approach of mesylation and azidation, as reported earlier [34,35].

**Scheme 5:**
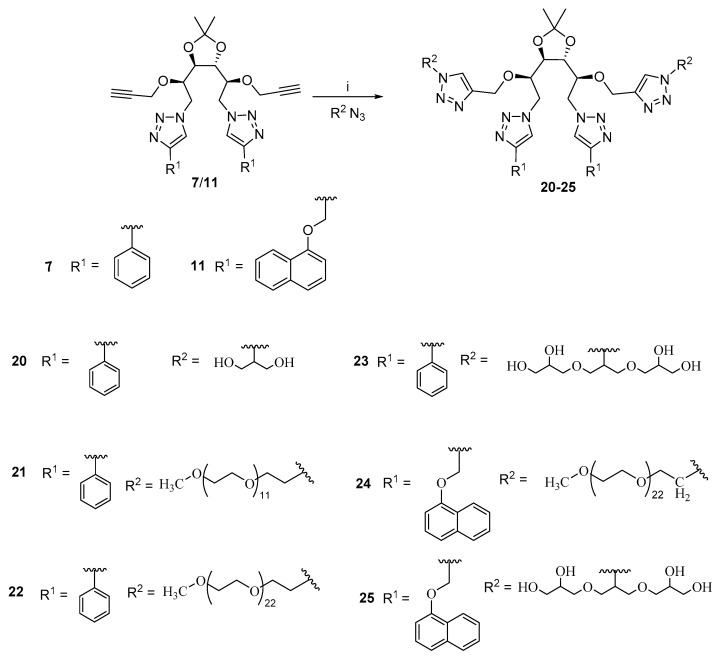
Synthesis of amphiphilies. (i) [Cu(PPh_3_)_3_]Br, DIPEA, DCM/DCM:DMF (1:1), 30 °C, 72 h.

Compounds **7/11** were grafted with hydrophilic glycerol azide (**14**)/triglyceryl azide (**17**)/monomethoxypolyethyleneglycol azides (**18** and **19**) on pendant acetylinc groups via the ‘click chemistry’ approach to yield compounds **20–25**. (Scheme 5).

The click coupling at both the pendant acetylinic groups was again confirmed by recording the IR spectrum of compounds **20–25**, in which no signal around 2100 cm^−1^ was observed, thereby confirming the absence of acetylinic and azide functionality. In addition, the structural confirmation of the final products was done by ^1^H and ^13^C NMR spectrum (Figures S10–S16) and HRMS analysis. The molecular weight of the PEG containing amphiphiles (**21**, **22**, and **24**) was determined using Gel Permeation Chromatography (Figure S17) and for amphiphiles **20**, **23**, and **25**.

### 3.2. Critical Aggregation Concentration (CAC) Measurement

**Figure 2:**
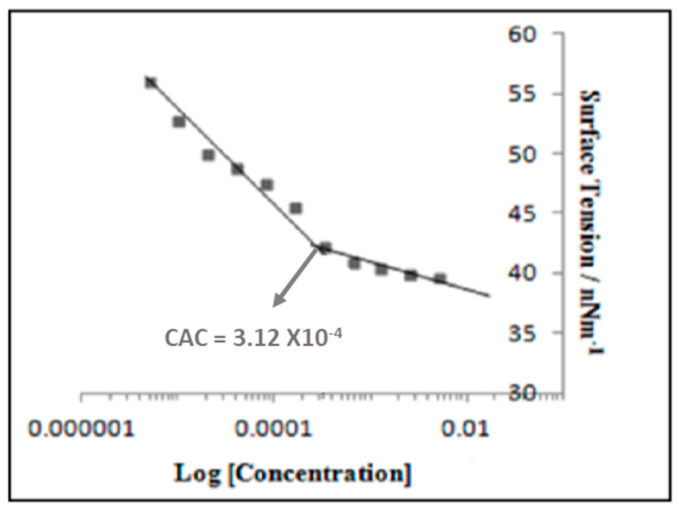
Plot of critical aggregation concentration (CAC) determination of amphiphile **24** using surface tension measurement.

All the synthesized amphiphiles except **20** have good aqueous solubility and were investigated for their self-assembling behavior in aqueous medium. Due to the existence of polar and non-polar groups in a single scaffold, amphiphiles tend to self-assemble beyond a certain minimum concentration referred to as the critical aggregation concentration (CAC) of the amphiphile. The CAC for the synthesized amphiphiles was determined using the pendant drop method based on surface tension measurement. An amphiphile stock solution of 5 mg mL^−1^ concentration was prepared and sequentially diluted to obtain a range of concentrations. The surface tension values were recorded at each concentration, and it was found that after a certain concentration, there was little change. Surface tension values were plotted against the log [concentration of amphiphile] and the point of curve break was referred to as the CAC of the amphiphile, as depicted in Figure 2. The amphiphilic systems with naphthyl group as the hydrophobic part formed relatively stable self-assemblies, as indicated by their slightly lower CAC values when compared with analogues constituted with phenyl groups (Table 1, Figure S18). The lowest CAC value was observed for amphiphile **24** having two monomethoxypolyethyleneglycol units (M_w_ = 1100 g/mol) and two naphthyl units grafted on the glucitol backbone.

### 3.3. Hydrophilic–Lipophilic Balance Determination

The hydrophilic–lipophilic balance (HLB) value is a measure of balance of size and strength of the hydrophilic and the lipophilic moieties of an amphiphilic molecule. The HLB value of the amphiphiles (**20–25**) has been calculated using the Griffin equation (Table 2) [36].

**Scheme 6:**
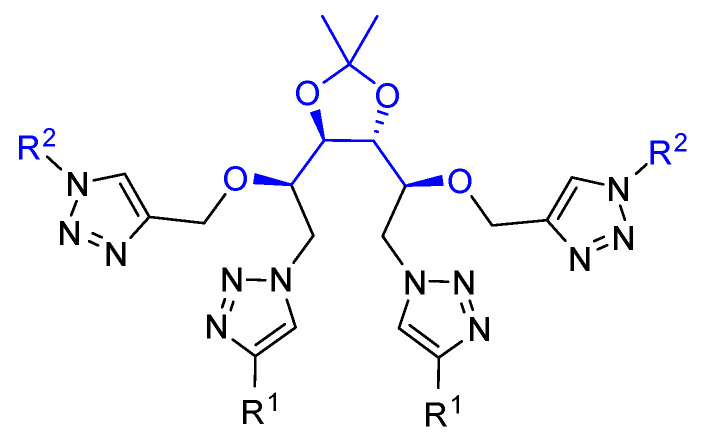
General structure of amphiphile.

HLB = 20 × M_h_/M

where in M_h_ is the molecular mass of the hydrophilic part (depicted in blue color in the general structure, Scheme 6) of the amphiphile, and M is the total molecular mass of the amphiphile. The low aqueous solubility of amphiphile **20** can be explained on the basis of a low HLB value.

### 3.4. Dynamic Light Scattering and Cryo-TEM Analysis

**Figure 3:**
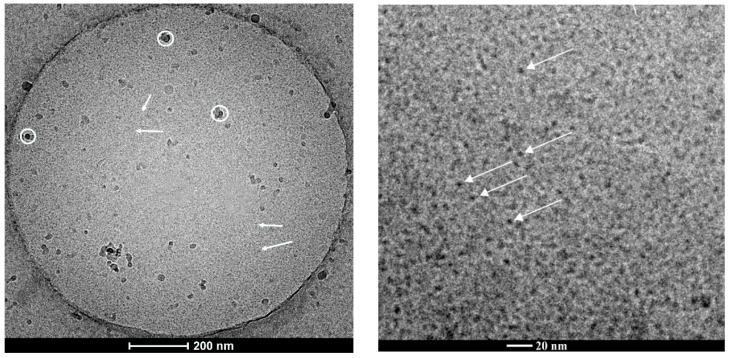
Cryo-TEM image of amphiphile **25** showing micellar assembly (indicated by arrows) and surface contamination (indicated by white circles) at different magnification.

Dynamic light scattering measurement was used for determining the size of the self-assembly. Cryo-TEM analysis was performed for one representative amphiphile (**25**) for getting an insight about the morphology of self-assembly formed by the amphiphilic construct. Both DLS and cryo-TEM studies were carried at a concentration of 5 mg mL^−1^, which is well above the CAC of the amphiphiles (Table 3 and Figure 3). DLS study generally reveals the hydrodynamic size of the particles, based on the assumption that the particles under investigation are spherical. The size distribution profile exhibited bimodal distribution in case of intensity and monomodal distribution in volume and number. The bimodality in intensity observed for the amphiphile **24**/**25** suggests the existence of two types of self-assemblies. An approximately similar particle size obtained from number and volume with the less intense peak in intensity indicated the predominance of these small size particles under aqueous conditions.

The image from cryo-TEM study for amphiphile **25** revealed particles in the size range of 5 nm depicted in the micrograph at two different magnifications (Figure 3) and was found to be in line with the DLS result.

### 3.5. Encapsulation Study

**Scheme 7:**
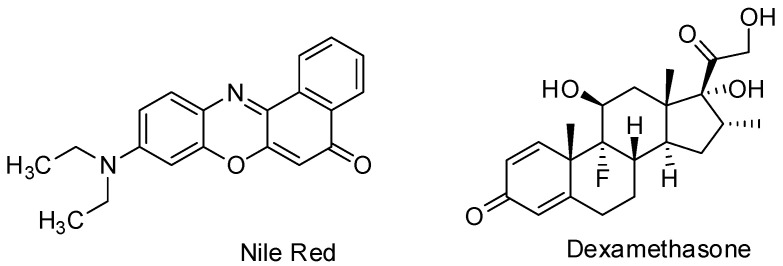
Structure of Nile Red and Dexamethasone.

To elucidate the applicability of the synthesized novel amphiphilic systems as nanocarriers for hydrophobic guests, a lipophilic solvatochromic dye Nile red and the drug dexamethasone were used as model hydrophobic guests Scheme 7. The encapsulation studies were carried out using the thin film method [37] i.e., a thin film of guest molecules was formed in the vial, and the solution of amphiphile in Milli-Q water was added.

#### 3.5.1. Nile Red Encapsulation Study

**Figure 4:**
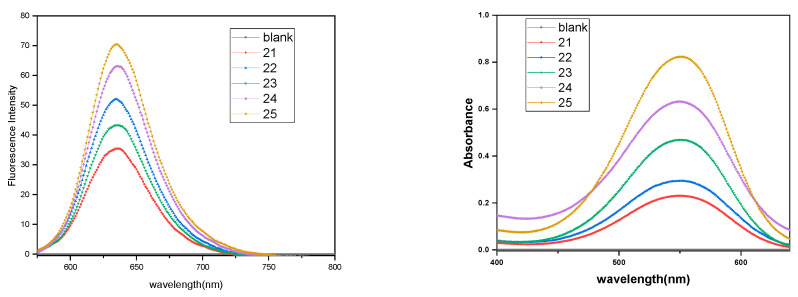
Emission spectra of Nile red encapsulated in the amphiphiles **21–25** in aqueous media and absorption spectra of Nile red encapsulated in the amphiphile solutions **21–25** in methanol.

**Figure 5:**
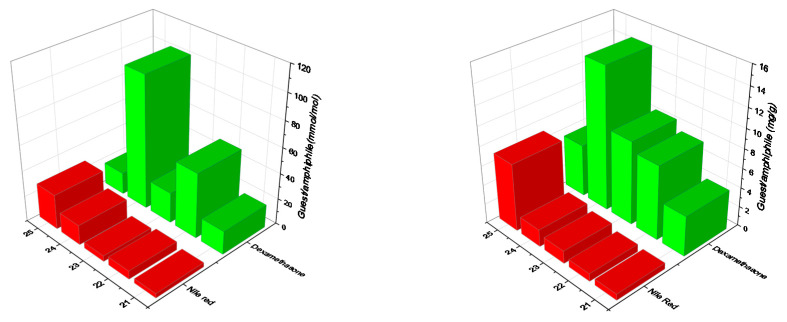
Guest loading capacity (mmol/mol) and guest loading efficiency (mg/g) of amphiphiles **21–25** for Nile red and dexamethasone.

Nile red is a solvatochromic dye with substantial fluorescence in a lipophilic microenvironment; first, it was used in the detection of intracellular lipid droplets by fluorescence microscopy. Nile red encapsulation was carried out by the thin film method. A thin film of Nile red was formed in the glass vial by adding 40 µL of 1 × 10^−1^ M stock solution to give 0.12 mg of Nile red per vial. Then, 5 mg mL^−1^ amphiphilic solution was added to the vial containing the dry film of Nile red and stirred at 1200 rpm for 12 h. The dye-encapsulated solution was filtered slowly using a 0.45 μm polytetrafluoroethylene (PTFE) filter. The encapsulation of Nile red in the amphiphiles could be inferred visually and from the fluorescence emission spectra of the Nile red containing amphiphilic solutions with almost no encapsulation seen with blank without any amphiphile (Figure 4). Thus, the filtrate was lyophilized and the residue was redissolved in anhydrous methanol for quantification. The absorbance spectra of encapsulated samples in methanol was recorded (Figure 4), the amount of encapsulated dye was calculated using Lambert–Beer Law, and the resulting loading capacity and loading efficiency are presented (Figure 5, Table 4).

#### 3.5.2. Dexamethasone Encapsulation

To illustrate the ability of glucitol-based amphiphilic constructs as nanocarriers for nonpolar guests, dexamethasone, an FDA-approved synthetic glucocorticoid used for its immune suppressant and anti-inflammatory effect, was encapsulated. The limiting factor in the usage of the above glucocorticoid drug is its low aqueous solubility. Therefore, enhancing the bioavailability of the drug using synthesized amphiphilic constructs will be of medicinal interest. Dexamethasone encapsulation was done using the thin film method with a dry film of 2.5 mg dexamethasone to which 5 mg mL^−1^ of amphiphile prepared in Millipore water was added, and the dispersion was allowed to stir for 22 h at 1200 rpm. The solution was filtered using a 0.45 μm PTFE filter, followed by lyophilizing the filtered solution, and it was redissolved in 1 mL acetonitrile:water mixture (2:3). The quantity of encapsulated dexamethasone was evaluated using an HPLC equipped with an internal UV absorption detector; measurements were done against a calibration curve using acetonitrile:water mixture (40:60) as the solvent system (Table 4, Figure 5, Figure S19).

It was observed that a higher loading efficiency of Nile red and dexamethasone in amphiphiles having a naphthyl unit over the amphiphiles having a phenyl unit (Figure 5, Table 4) was a result of favorable hydrophobic interactions (majorly π–π interactions) between the core of the self-assembly formed by the amphiphiles and hydrophobic guests, leading to their successful entrapment. The amphiphile **24** was observed to exhibit the highest loading for dexamethasone, whereas amphiphile **25** exhibited the highest loading for Nile red as compared to the other amphiphiles studied. This could be attributed to the structure-based interactions of the guests with the self-assembled amphiphilic systems. Nile red being more lipophilic than dexamethasone, as the latter has two hydroxyls and one carboxyl group present, shows preferential encapsulation by the amphiphile **25** having a lower HLB value (9.72) (Table 2). However, on the other hand, dexamethasone being more hydrophilic is expected to be entrapped more efficiently by the amphiphile that has a relatively higher HLB value (Table 2). Although the HLB value of amphiphiles **22** and **24** is higher and comparable i.e., 16.62 and 15.74 (Table 2) respectively, but the naphthalene-based amphiphile **24** exhibits higher encapsulation as compared to the phenyl moiety containing amphiphile **22**. This is probably due to more favorable π–π interactions between amphiphile **24** and dexamethasone as compared to analogue **22** constituted from the phenyl ring. The order of loading capacity for dexamethasone was observed to be **24** > **22** > **23** > **21** > **25**.

### 3.6. Cytotoxicity Study

**Figure 6:**
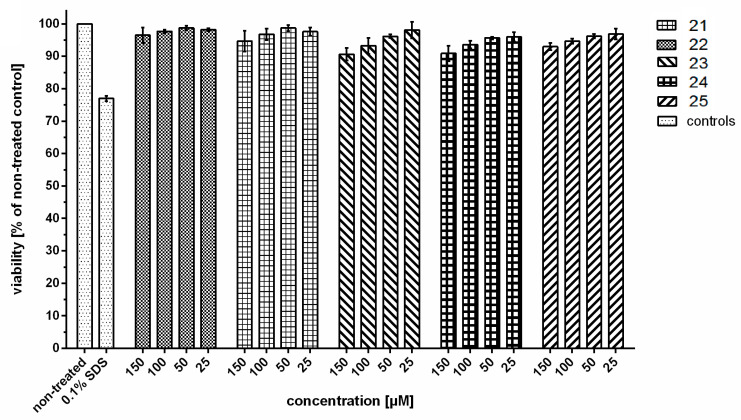
Cytotoxicity profile of amphiphiles **21–25** determined by 3-(4,5-dimethylthiazol-2-yl)-5-(3-carboxymethoxyphenyl)-2-(4-sulfophenyl)-2*H*-tetrazolium (MTS) assay in A549 cells 24 h post treatment. Each bar represents the mean value of three independent experiments (*n* = 3) measured in triplicate with SEM.

Considering the fact that the major requirement of a carrier for biomedical applications is that it should be biocompatible and non-cytotoxic, we evaluated the in vitro cytotoxicity of the synthesized amphiphilic systems except **20** due to its poor water solubility using A549 cells (adenocarcinomic human alveolar basal epithelial cells) (Figure 6). The A549 cells were treated with the compounds **21–25** at a concentration of 150, 100, 50, and 25 μM, and the cell viability was assessed using MTS assay after 24 h.

The cytotoxicity profile suggested the synthesized amphiphilic systems to be non-cytotoxic even up to the highest test concentration 150 μm. Furthermore, it has been observed that the amphiphilic systems constituted from the phenyl moiety and mPEG (Mw = 1100 g/mol) have better biocompatibility as compared to the naphthyl and triglycerol-based amphiphilic architectures, which exhibit marginal cytotoxicity.

## 4. Conclusions

We have successfully utilized *D*-glucitol-3,4-diacetonide-5,6-diazide as a novel core for developing a small library of non-ionic amphiphilic constructs. The synthesis has been accomplished by grafting different hydrophobic and hydrophilic groups on the *D*-glucitol core via an atom economical click approach. Surface tension measurements and dynamic light scattering studies revealed that five out of six amphiphilic architectures tend to form supramolecular aggregates in aqueous solution with CAC values in the order of 10^−4^ M and particle size in the range of 5–200 nm. The self-assemblies thus formed have been efficaciously explored for the encapsulation of guests. Amphiphiles containing the naphthyl moiety were found to be superior in comparison to the phenyl group containing amphiphiles for loading both Nile red and dexamethasone. Amphiphile **25** consisting of naphthyl and dendritic triglycerol exhibited the highest loading capacity for Nile red. However, in the case of dexamethasone, the amphiphilic construct **24** built using mPEG and a naphthyl unit exhibited a maximum loading capacity. Interestingly, the evaluation of cytotoxicity profile performed revealed the biocompatibility of the synthesized amphiphilic architectures at all the tested concentrations. To the best of our knowledge, this is the first report where glucitol diazide has been used as a core for developing amphiphilic constructs which have been efficaciously explored for their guest encapsulation capacity. This study opens a gateway for the development of amphiphiles for fabricating interesting nanostructures using a *D*-glucitol derivative.

## Figures and Tables

**Table 1:** CAC of amphiphiles determined using surface tension method.

Amphiphile	CAC (M)
**21**	3.75 × 10^−3^
**22**	9.25 × 10^−4^
**23**	6.57 × 10^−4^
**24**	3.12 × 10^−4^
**25**	6.25 × 10^−4^

**Table 2:** Hydrophilic–lipophilic balance (HLB) value of amphiphiles (**20–25**) determined using Griffin equation [35].

Amphiphile	Composition		HLB
	R^1^	R^2^	
**20**	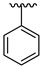	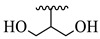	7.83
**21**	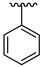	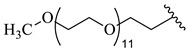	14.49
**22**	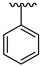	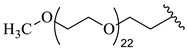	16.62
**23**	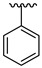	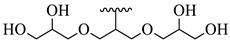	11.16
**24**	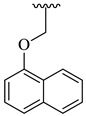	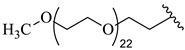	15.74
**25**	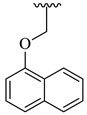	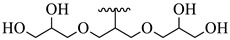	9.72

**Table 3:** Particle size distribution profile of amphiphile self-assemblies above the CAC in aqueous solution as measured by dynamic light scattering (DLS) at 25 °C.

Amphiphile	DLS (*D*_h_, nm)		
	I	V	N
**21**	147	93	51
**22**	138	75	39
**23**	94	46	30
**24**	15 279	12	10
**25**	6 166	5	4

**I**: Intensity distribution; **V**: Volume distribution; **N**: Number distribution; DLS data recorded at 5 mg mL^−1^.

**Table 4:** Loading capacity and loading efficiency of amphiphiles **21–25**.

Amphiphile	Loading Capacity (Guest/Amphiphile) (mmol/mol)		Loading Efficiency (Guest/Amphiphile) (mg/g)	
	Nile red	Dexamethasone	Nile Red	Dexamethasone
**21**	3.46	18.25	0.64	4.16
**22**	6.53	50.91	0.8	7.68
**23**	4.48	23.84	1.32	8.64
**24**	15.44	103.59	1.78	14.72
**25**	27.2	17.22	6.97	5.44

## Supplementary Materials

The following are available online at https://www.mdpi.com/2073-4360/12/6/1421/s1. Figure S1: ^1^H and ^13^C NMR spectra of compound **3** in DMSO-*d*_6_, Figure S2: ^1^H and ^13^C NMR spectra of compound **5** in CDCl_3_, Figure S3: Overlay of ^13^C NMR of compounds **3** and **5** (top) and ^13^C and DEPT-135 spectrum of compound **5** (bottom), Figure S4: ^1^H and ^13^C NMR spectra of compound **6** in CDCl_3_, Figure S5: 2D HETCOR spectrum for compound **6** in CDCl_3_, Figure S6: ^1^H and ^13^C NMR spectra of compound **10** in CDCl_3_, Figure S7: ^1^H and ^13^C NMR spectra of compound **7** in CDCl_3_, Figure S8: 2D HETCOR spectrum of compound **7** in CDCl_3_, Figure S9: ^1^H and ^13^C NMR spectra of compound **11** in CDCl_3_, Figure S10: ^1^H and ^13^C NMR spectra of compound **20** DMSO-*d*_6_, Figure S11: 2D HETCOR spectrum of compound **20** in DMSO-*d*_6_, Figure S12: ^1^H and ^13^C NMR spectra of compound **21**, Figure S13: ^1^H and ^13^C NMR spectra of compound **22**, Figure S14: ^1^H and ^13^C NMR spectra of compound **23**, Figure S15: ^1^H and ^13^C NMR spectra of compound **24**, Figure S16: ^1^H and ^13^C NMR spectra of compound **25**, Figure S17: Gel permeation chromatogram of amphiphiles (A) 21 (B) 22 (C) 24, Figure S18: Critical aggregation concentration (CAC) of amphiphiles (A) 21 (B) 22 (C) 23 (D) 25 in aqueous solution by surface tension measurements at 25 °C, Figure S19: Calibration curve of Dexamethasone using HPLC and representative HPLC chromatogram of dexamethasone encapsulated samples (A) 21(B) 22 (C) 24.
