# Supplementary material for: Chemoenzymatic Synthesis of D-Glucitol-Based Non-Ionic Amphiphilic Architectures as Nanocarriers
Source: Polymers (Basel). 2020 Jun 25;12(6):1421. doi: 10.3390/polym12061421 (PMC7362011; doi:10.3390/polym12061421)
Supplement: Supplementary file 1 [file polymers-12-01421-s001.pdf]

Electronic Supplementary Information

Chemo-enzymatic Synthesis of *D*-Glucitol Based Non-ionic Amphiphilic Architectures as Nanocarriers

Priyanka Manchanda,[1] Katharina Achazi,[2] Diksha Verma,[1] Christoph Böttcher,[3] Rainer Haag,[*4] Sunil K. Sharma[1,*]

[1]*Department of Chemistry, University of Delhi, Delhi-110 007*
[2]*Institut für Chemie und Biochemie, Freie Universität Berlin, Arnimallee 22, 14195 Berlin, Germany*
[3]*Forschungszentrum für Elektronenmikroskopie, Institut für Chemie und Biochemie, Freie Universität Berlin, Fabeckstraße 36a, 14195 Berlin, Germany*
[4]*Institut für Chemie und Biochemie, Freie Universität Berlin, Takustraße 3, 14195 Berlin, Germany*

List of Contents

1. Chemo-enzymatic synthesis of Glyeryl azide (2-azidopropane-1,3-diol) (**14**) 2
2. **Figure S1**. ^{1}H &^{13C} NMR spectra of compound **3** 3
3. **Figure S2**. ^{1}H &^{13}C NMR spectra of compound **5** 4
4. **Figure S3**. Overlay of ^{13}C NMR of compounds **3** and **5** (top) and ^{13}C and DEPT-135 spectrum of compound **5** (bottom). 5
5. **Figure S4.** ^{1}H &^{13}C NMR spectra of compound **6** 6
6. **Figure S5.** ^{2}D HETCOR spectrum of compound **6** 7
7. **Figure S6.** ^{1}H &^{13}C NMR spectra of compound **10** 8
8. **Figure S7.** ^{1}H &^{13}C NMR spectra of compound **7** 9
9. **Figure S8.** ^{2}D HETCOR spectrum of compound **7** 10
10. **Figure S9.** ^{1}H & ^{13}C NMR spectrum of compound **11** 11
11. **Figure S10.** ^{1}H &^{13}C NMR of amphiphile **20** 12
12. **Figure S11.** ^{2}D HETCOR spectrum of amphiphile **20** 13
13. **Figure S12.** ^{1}H &^{13}C NMR spectra of amphiphile **21** 14
14. **Figure S13.** ^{1}H &^{13}C NMR spectra of amphiphile **22** 15
15. **Figure S14.** ^{1}H &^{13}C NMR spectra of amphiphile **23** 16
16. **Figure S15.** ^{1}H &^{13}C NMR spectra of amphiphile **24** 17
17. **Figure S16.** ^{1}H &^{13}C NMR spectra of amphiphile **25** 18
18. **Figure S17.** Gel permeation chromatogram of amphiphiles (A) **21** (B) **22** (C) **24**. 19
19. **Figure S18.** Critical aggregation concentration (CAC) of amphiphiles (A) **21** (B) **22** (C) **23** (D) **25** in aqueous solution by surface tension measurements at 25 ^{o}C. 20
20. **Figure S19.** Calibration curve of Dexamethasone using HPLC and representative HPLC chromatogram of dexamethasone encapsulated samples. 21

Chemo-enzymatic synthesis of **Glyceryl azide (**2-azidopropane-1,3-diol) (**14**)

The compound **14** was synthesized in four steps by following a chemo-enzymatic approach reported earlier from our group (**Scheme 2**).[1] A homogenous mixture of glycerol (10 g, 108. 69 mmol) and vinyl acetate (25 mL, 2.5 equivalent, 271.45 mmol) in THF (200 mL) were placed in a round bottom flask. Immobilized enzyme *Candida antarctica* lipase (Novozyme-435, 3.3 g, 10 wt. % of reactants) was then added and the resultant mixture was stirred for 1 h at 25 ^{o}C. After that the enzyme was filtered off and the filtrate was concentrated under reduced pressure to give 2-hydroxypropane-1,3-diyl diacetate (**12**) as a colourless viscous liquid in 93% yield. To the solution of compound **12** (5g, 1 equivalent, 28.40 mmol**)** in dichloromethane (150 mL) at 0 ^{o}C, methanesulfonyl chloride (6.5 mL, equivalent, 85.22 mmol) and triethylamine (11.6 mL, 3 equiv., 85.22 mmol) was added. After completion of the reaction, the precipitate was removed by filtration and the mixture concentrated under vacuum to give mesylated product. The crude product so obtained was then dissolved in anhydrous DMF (200 mL) and sodium azide (9.23 g, 5 equivalent, 142.20 mmol) was added. The reaction mixture was stirred at 90 ^{o}C under argon for 5 h. After completion of the reaction DMF was removed under reduced pressure using rotary evaporator to obtain compound **13**. The crude product (**13**) was purified by column chromatography. For deacetylation, compound **13** was taken in ethanol (200 mL) and potassium carbonate (17.16 g) was then added and the reaction mixture stirred at room temperature for 24 h. Progress of the reaction was monitored by TLC (methanol: chloroform). After completion, the precipitate was removed by filtration and the mixture was concentrated under vacuum. The compound was purified by column chromatography using silica gel to obtain pure 2-azidopropane-1,3-diol (**14**) as a colourless viscous liquid in 80% yield. **^{1}H NMR (400 MHZ, Acetone-d$_6$) δ:** 4.27-4.24 (t, 2H, OH), 3,71-3.55 (4H, multiplets of doublet), 3,52-3.46 (m, 1H). **^{13}C NMR (100.5 MHz, Acetone-d$_6$) δ:**65.93 and 62.32. **IR** (**KBr**)□**$_{max}$(cm^{-1}**): 3351.6, 2945.2, 2933.1, 2109.1, 1451.3, 1336.2, 1254.3, 1037.7 and 1010.8 cm^{-1}.

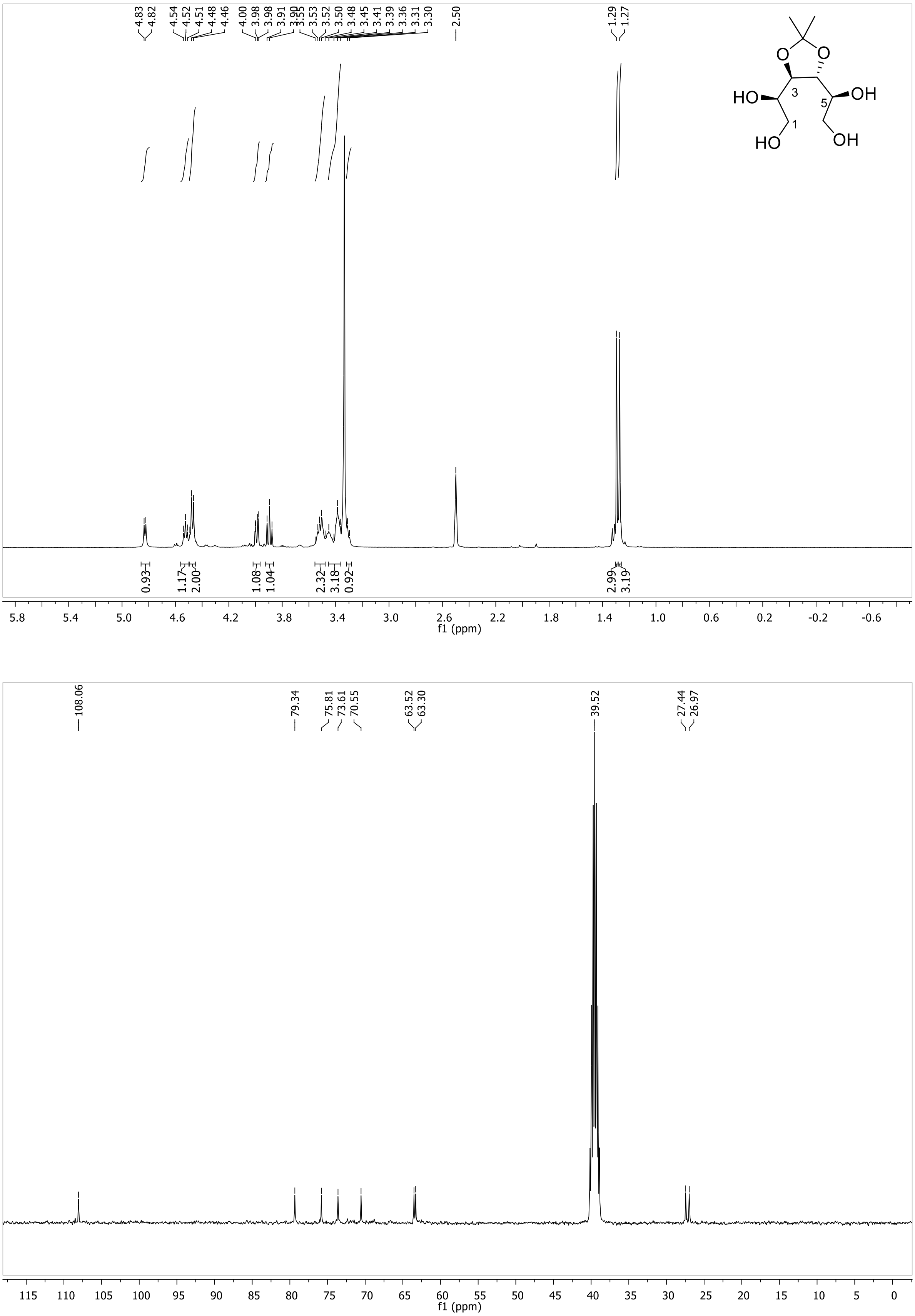

Figure S1.^{1}H and ^{13}C NMR spectra of compound **3** in DMSO-d_6.

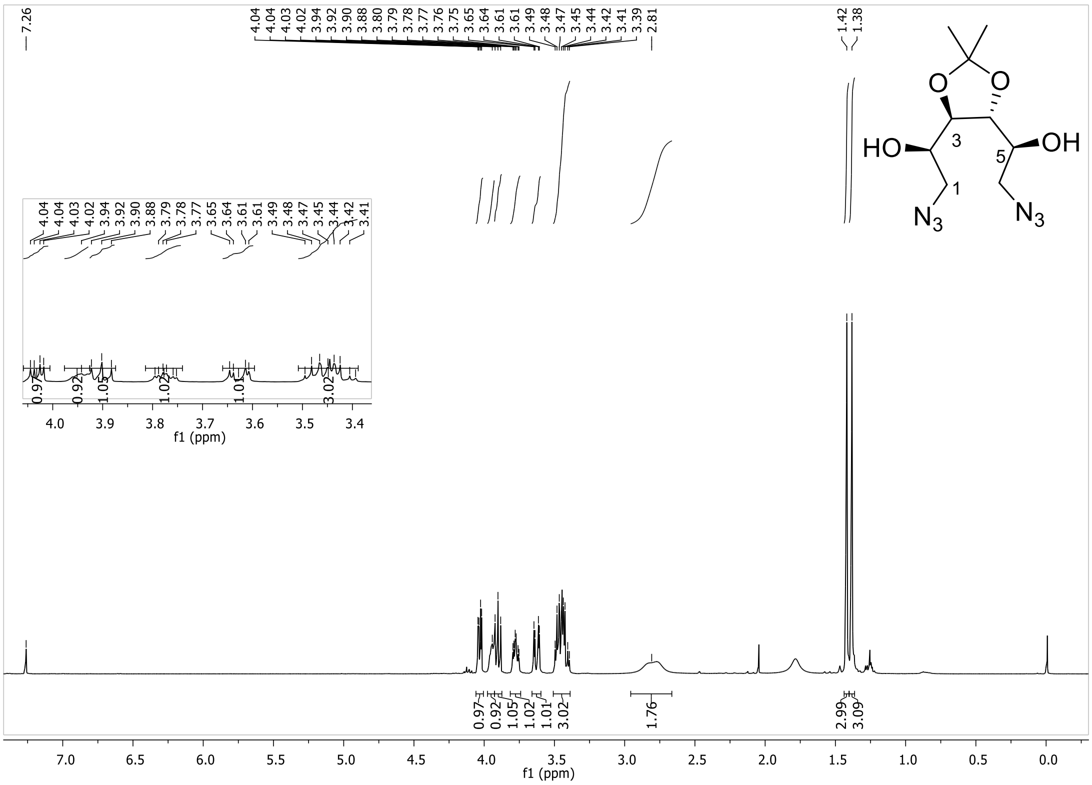

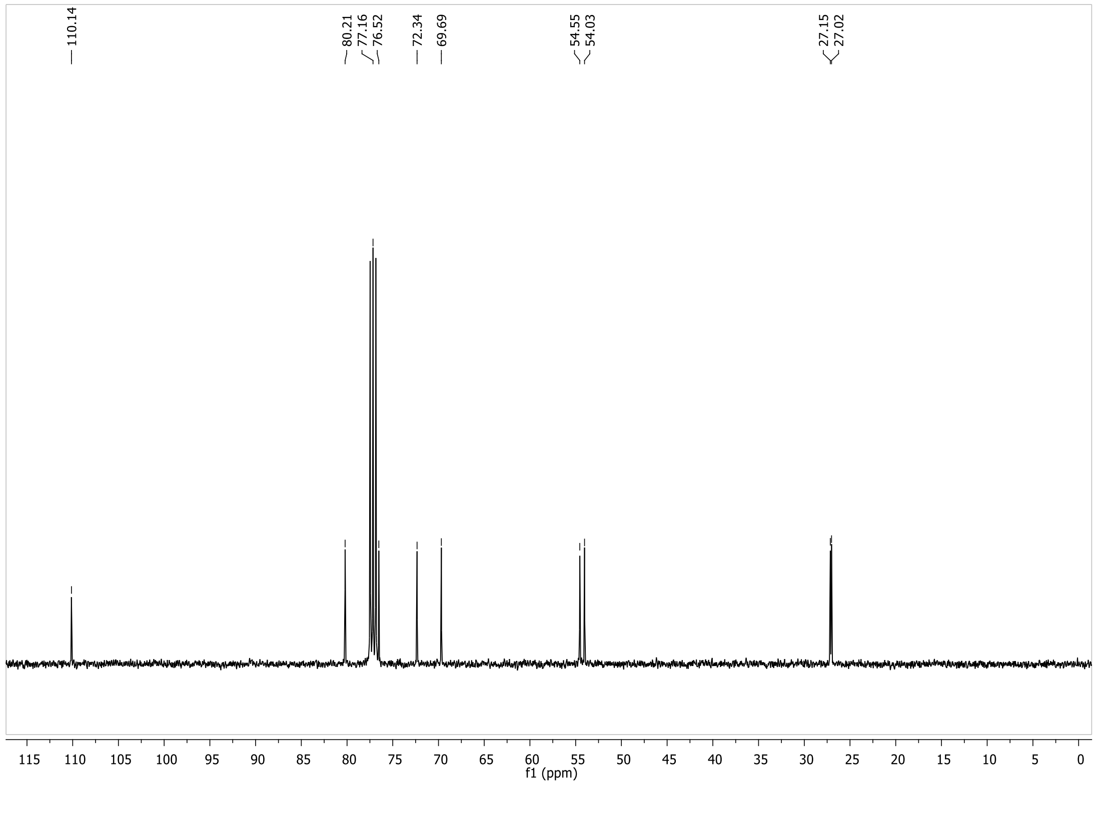

Figure S2.^{1}H and ^{13}C NMR spectra of compound **5** in $CDCl_3$.

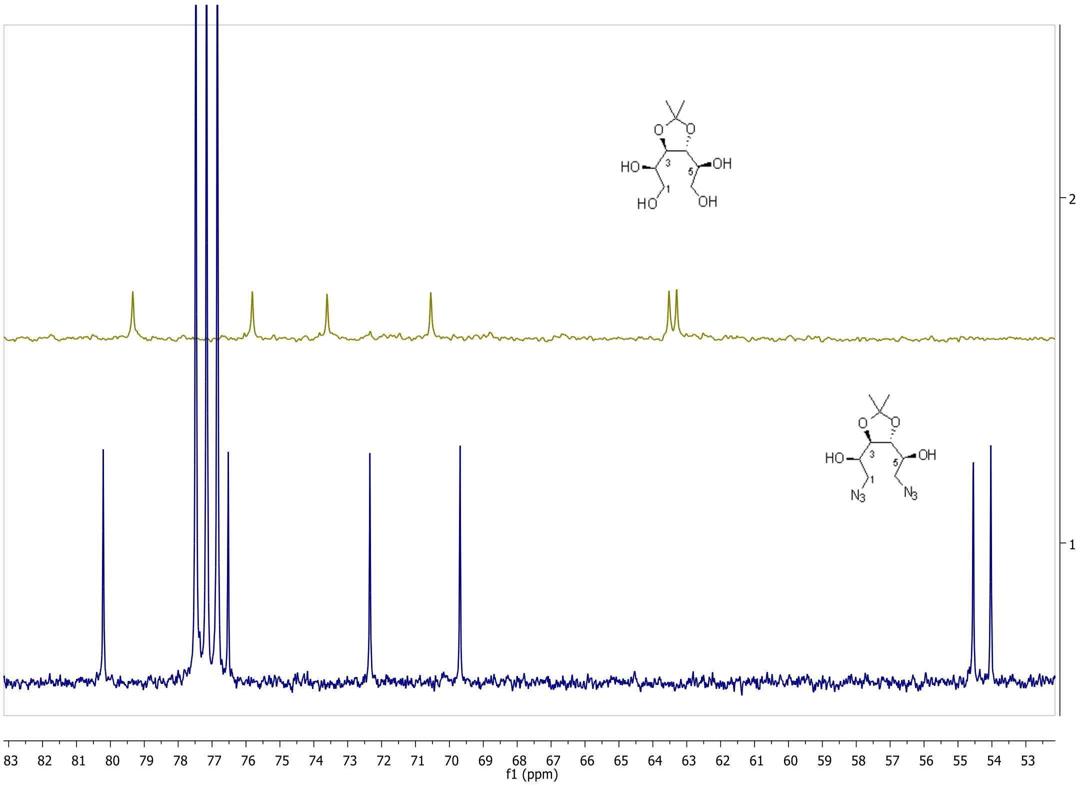

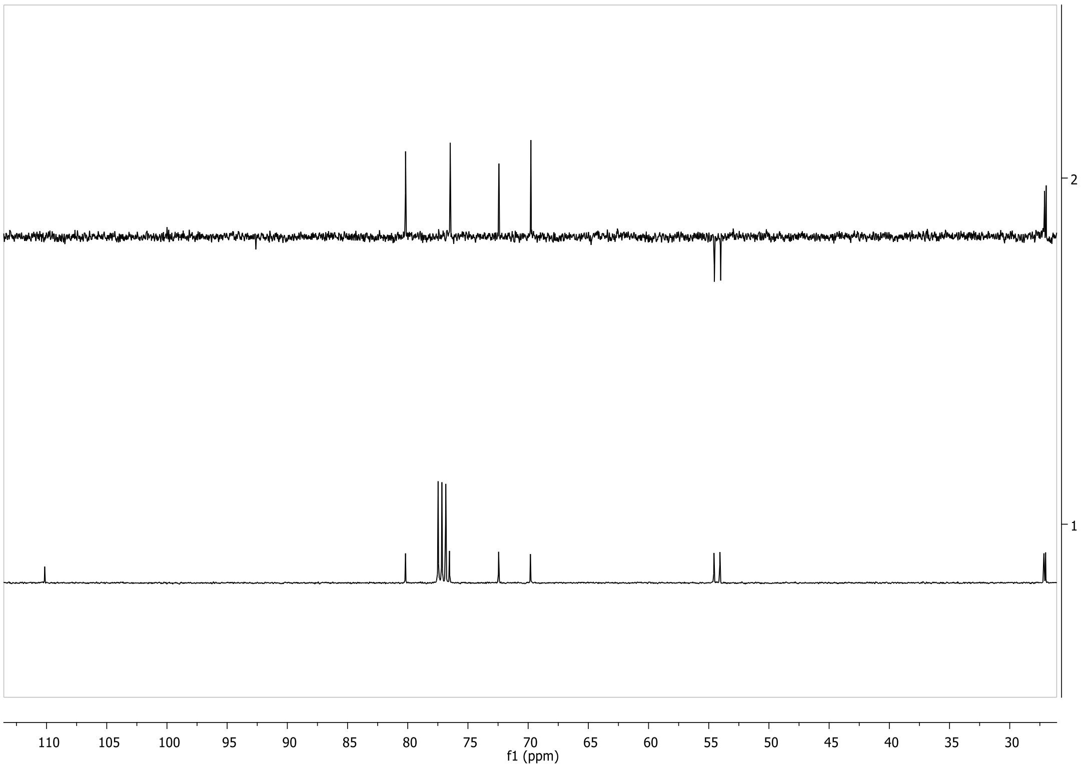

Figure S3. Overlay of ^{13}C NMR of compounds **3** and **5** (top) and ^{13}C and DEPT-135 spectrum of compound **5** (bottom).

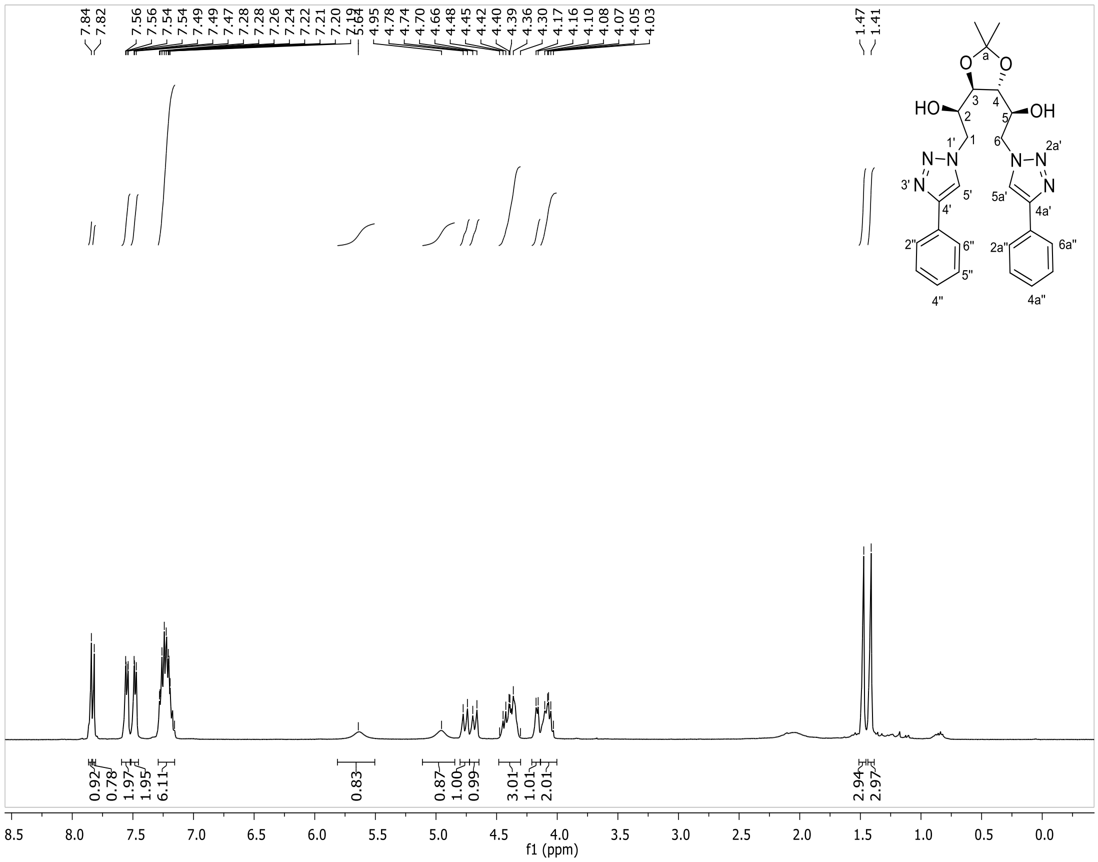

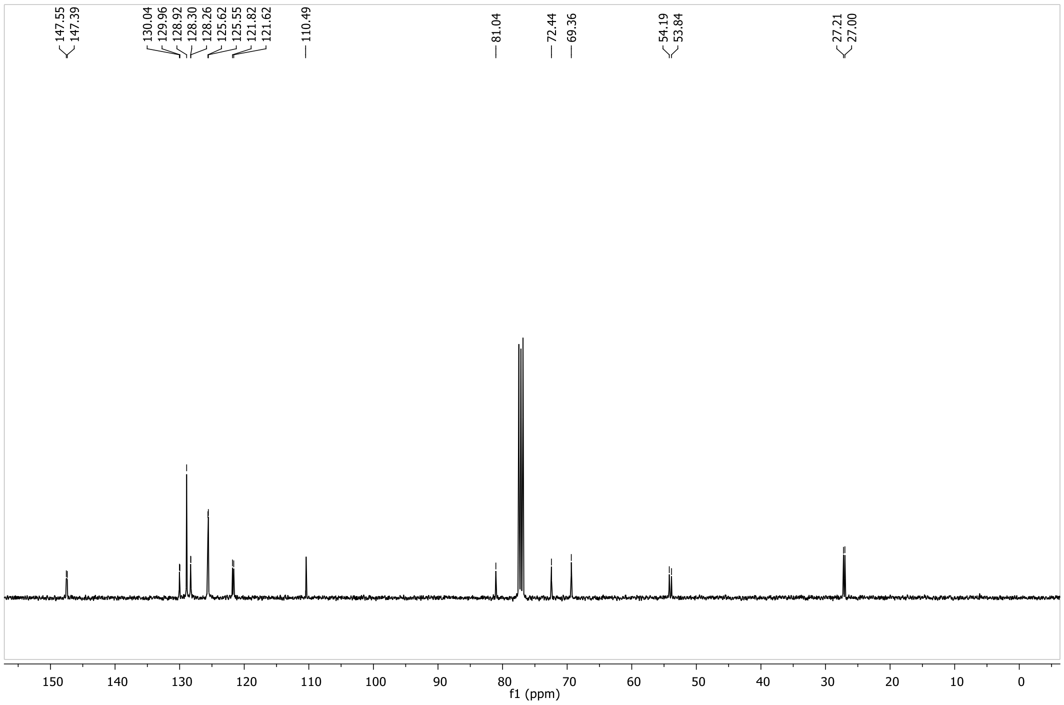

Figure S4. ^{1}H and ^{13}C NMR spectra of compound **6** in $CDCl_3$.

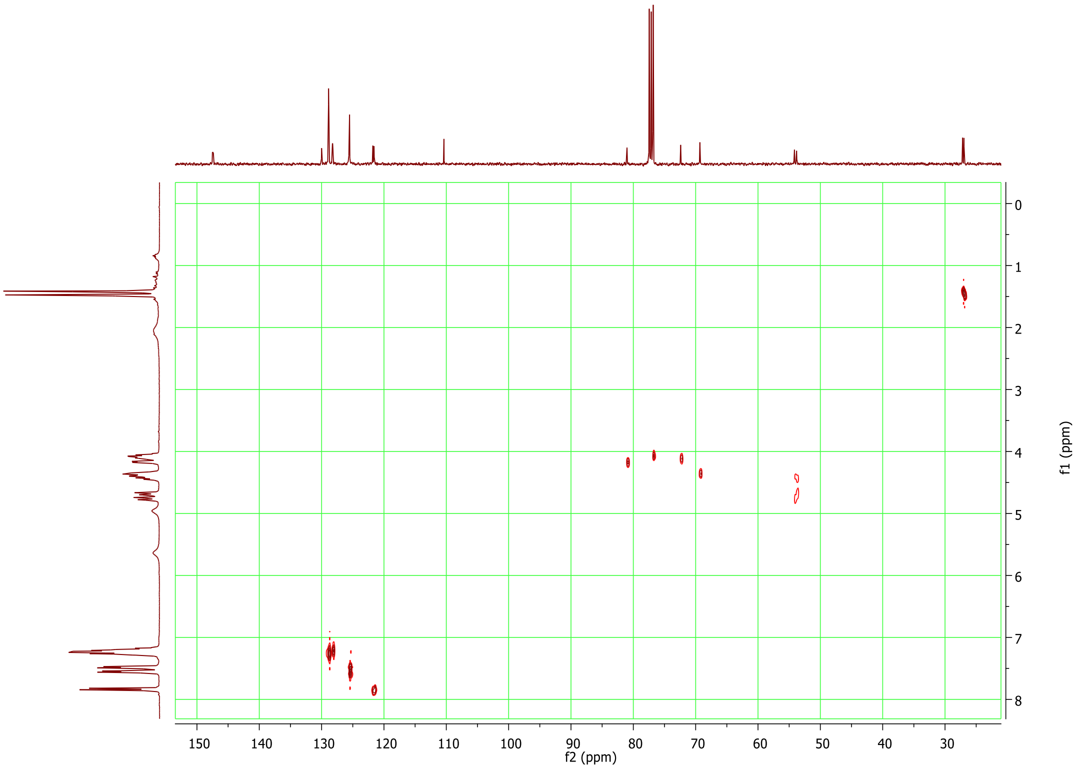

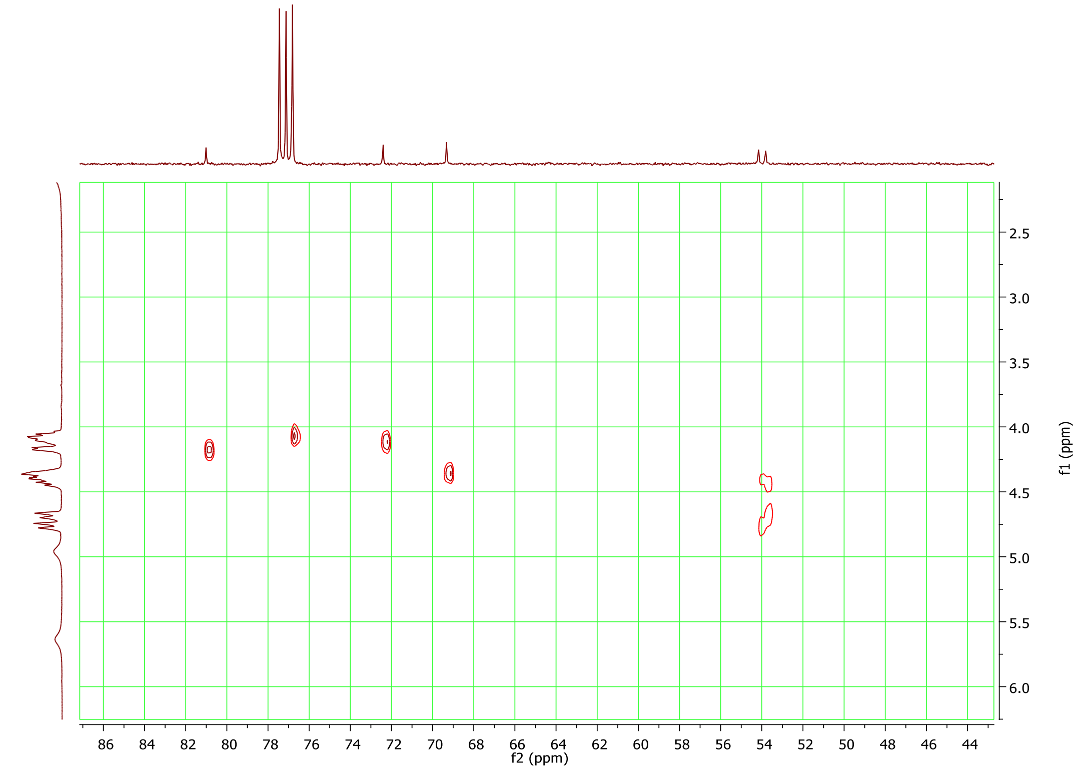

Figure S5. 2D HETCOR spectrum for compound **6** in $CDCl_3$.

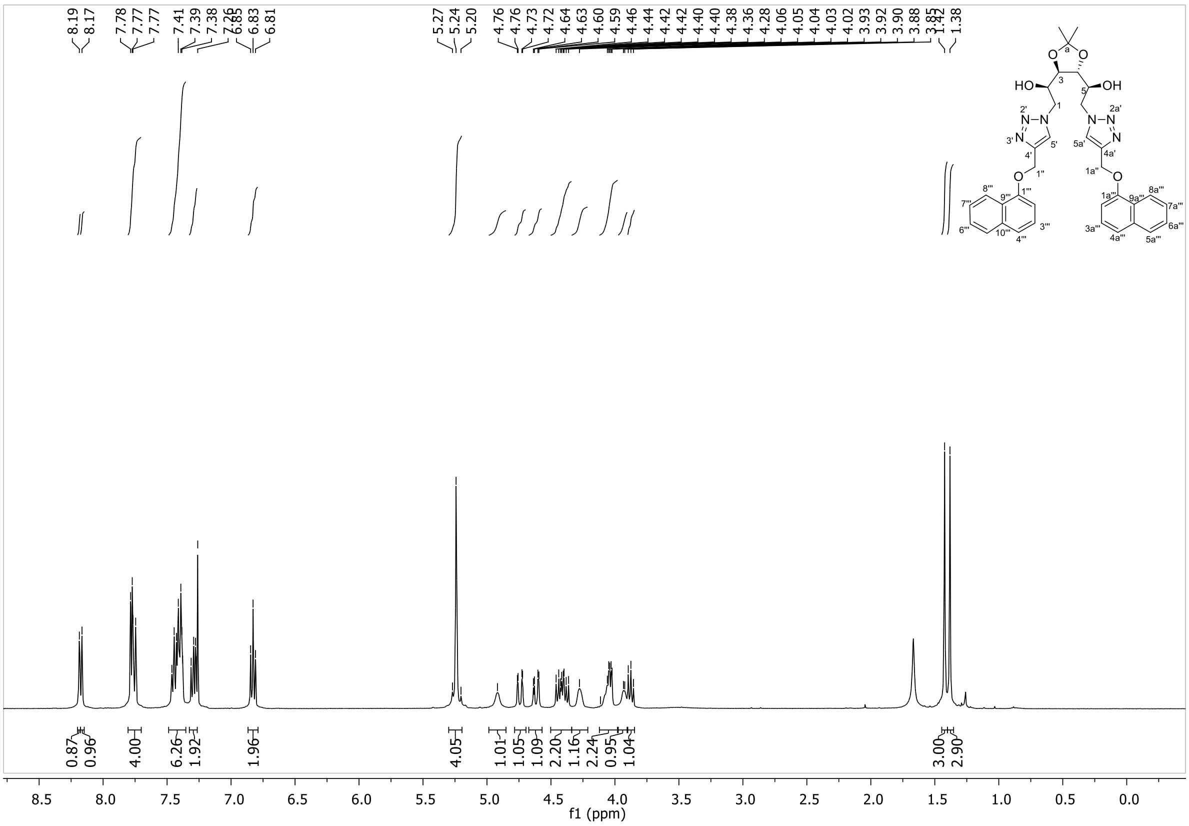

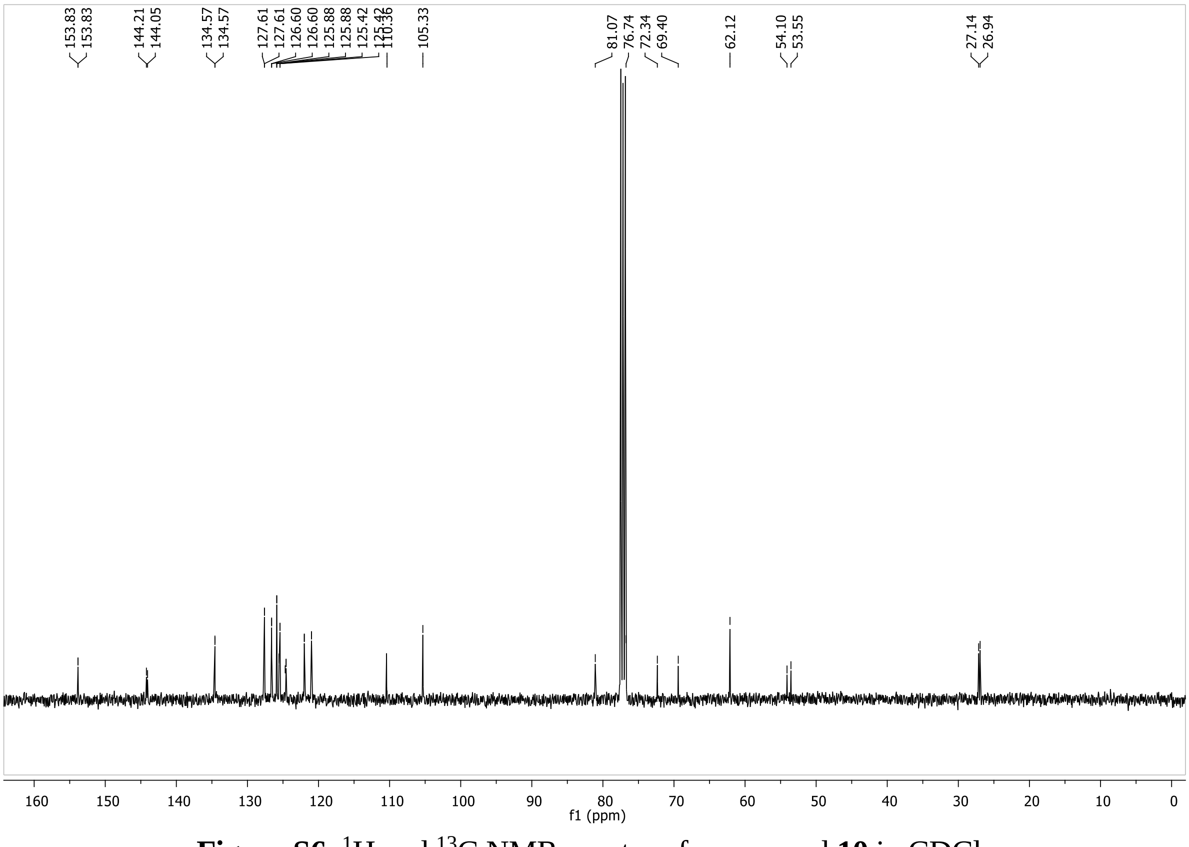

Figure S6. ^{1}H and ^{13}C NMR spectra of compound **10** in $CDCl_3$

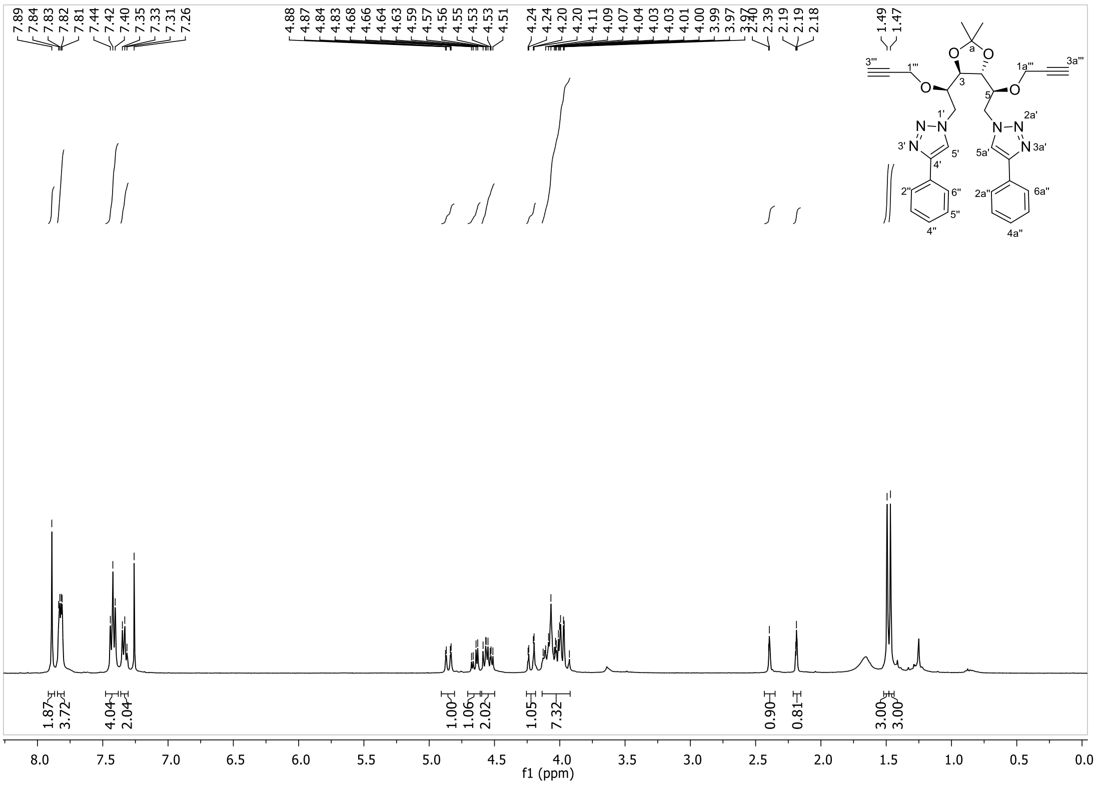

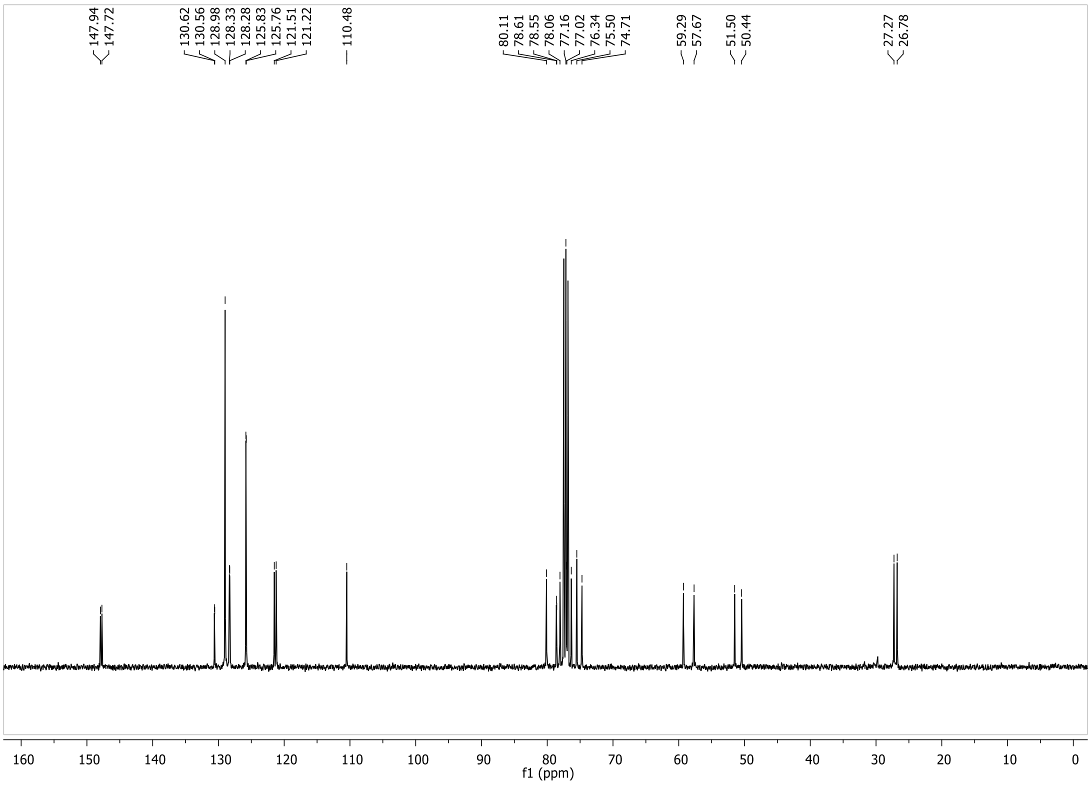

Figure S7. ^{1}H and ^{13}C NMR spectra of compound **7** in $CDCl_3$.

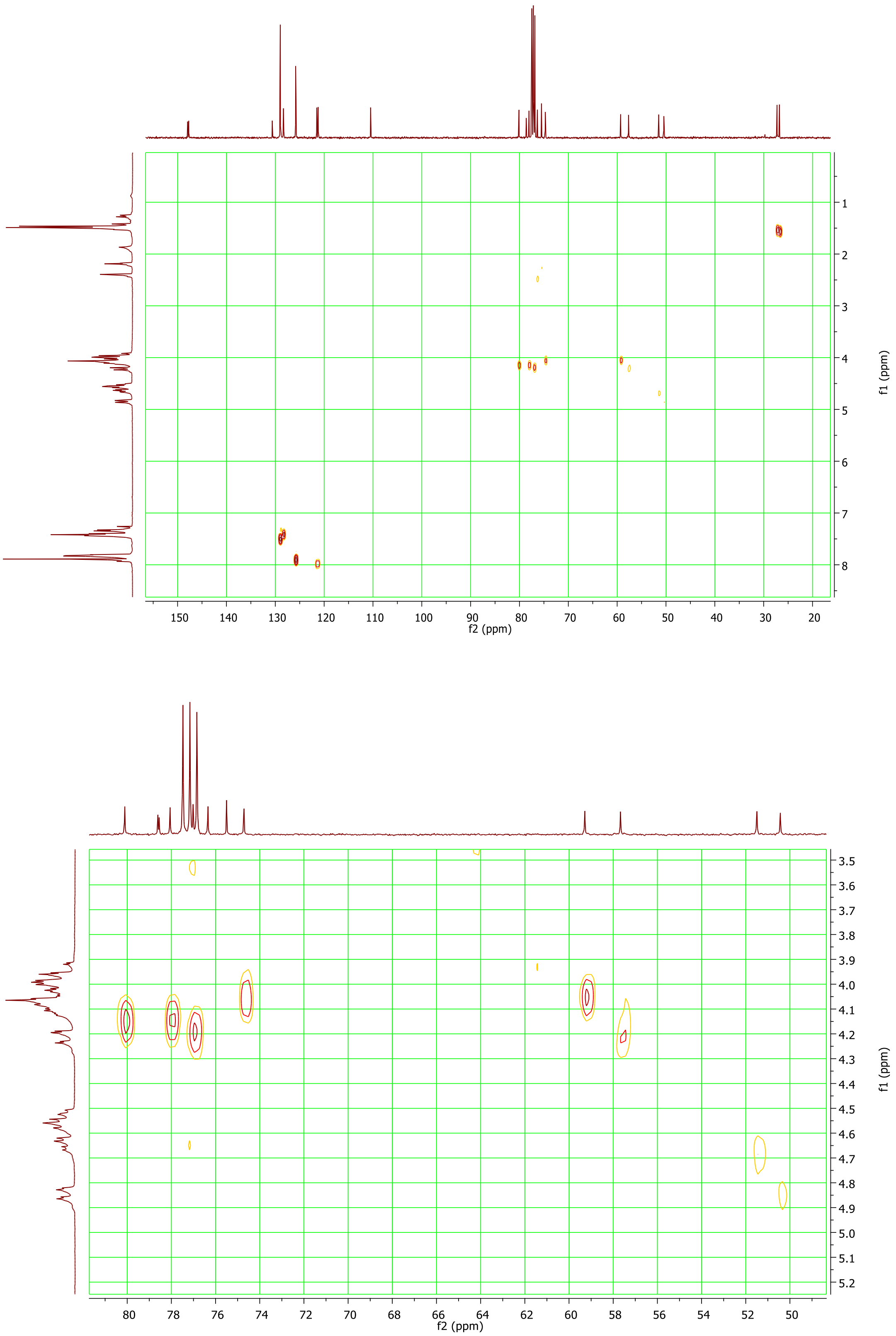

Figure S8. [2]D HETCOR spectrum of compound **7** in $CDCl_3$.

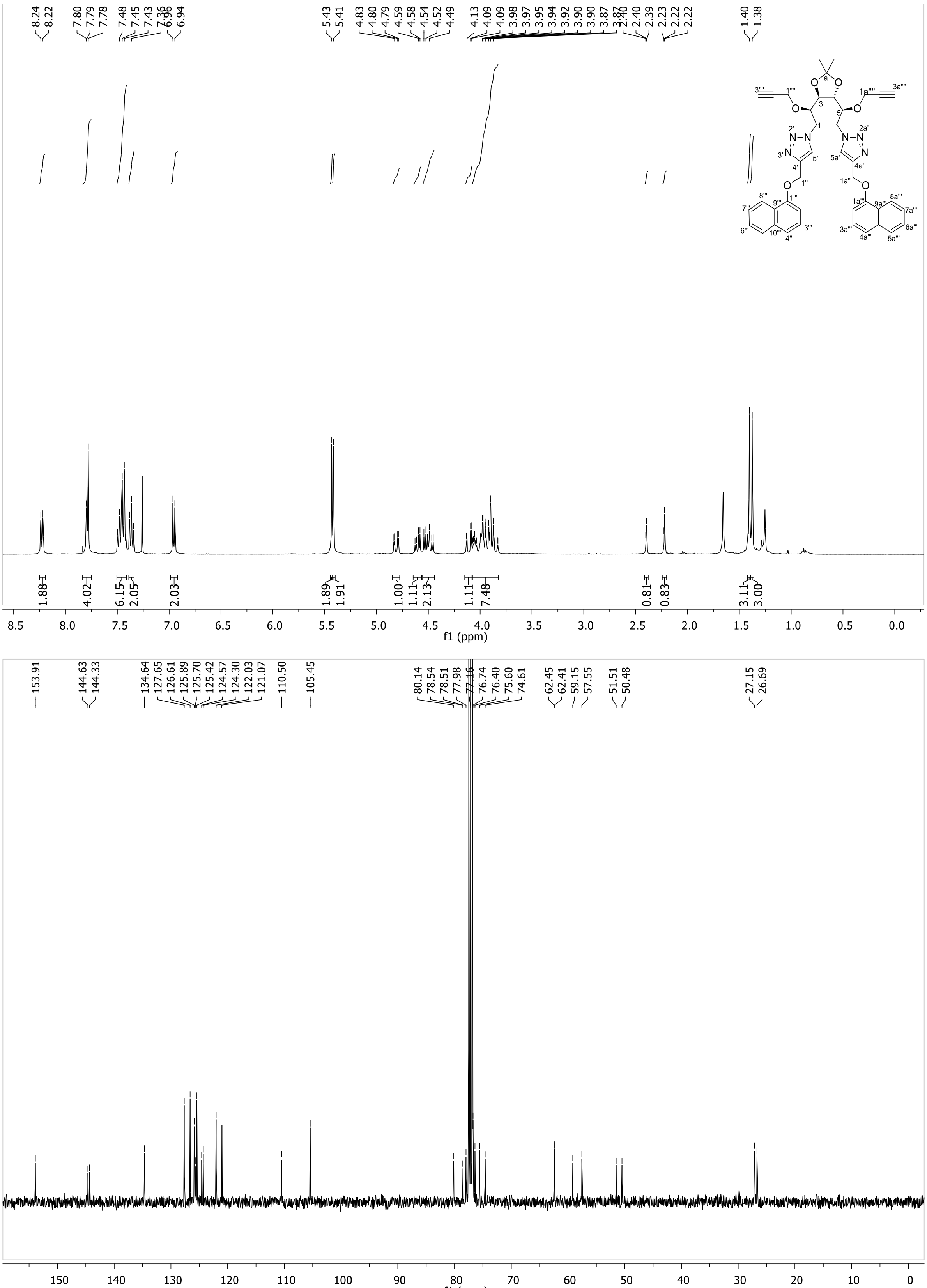

Figure S9. ^{1}H and ^{13}C NMR spectra of compound **11** in $CDCl_3$.

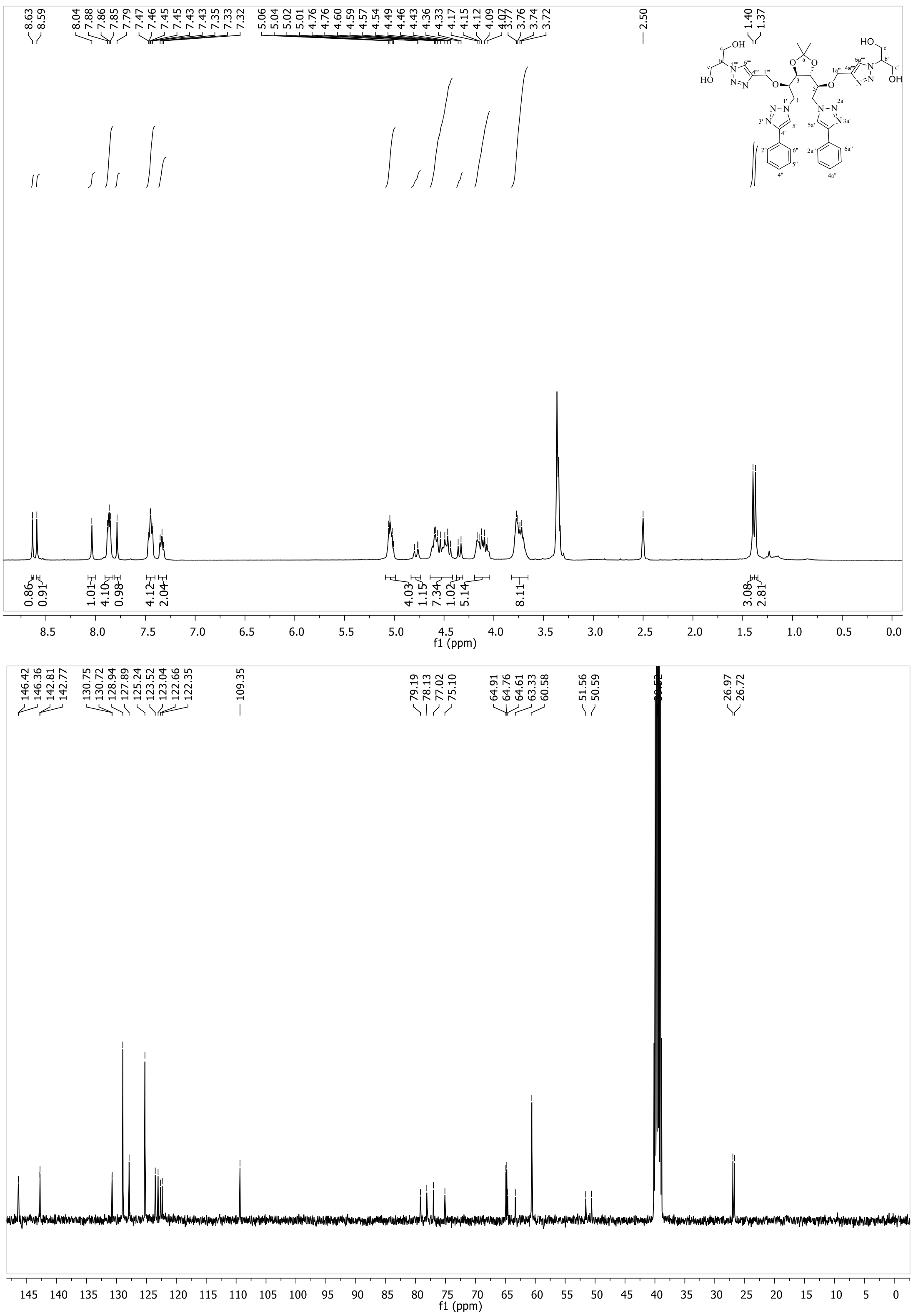

Figure S10. ^{1}H and ^{13}C NMR spectra of compound **20** DMSO-d_6

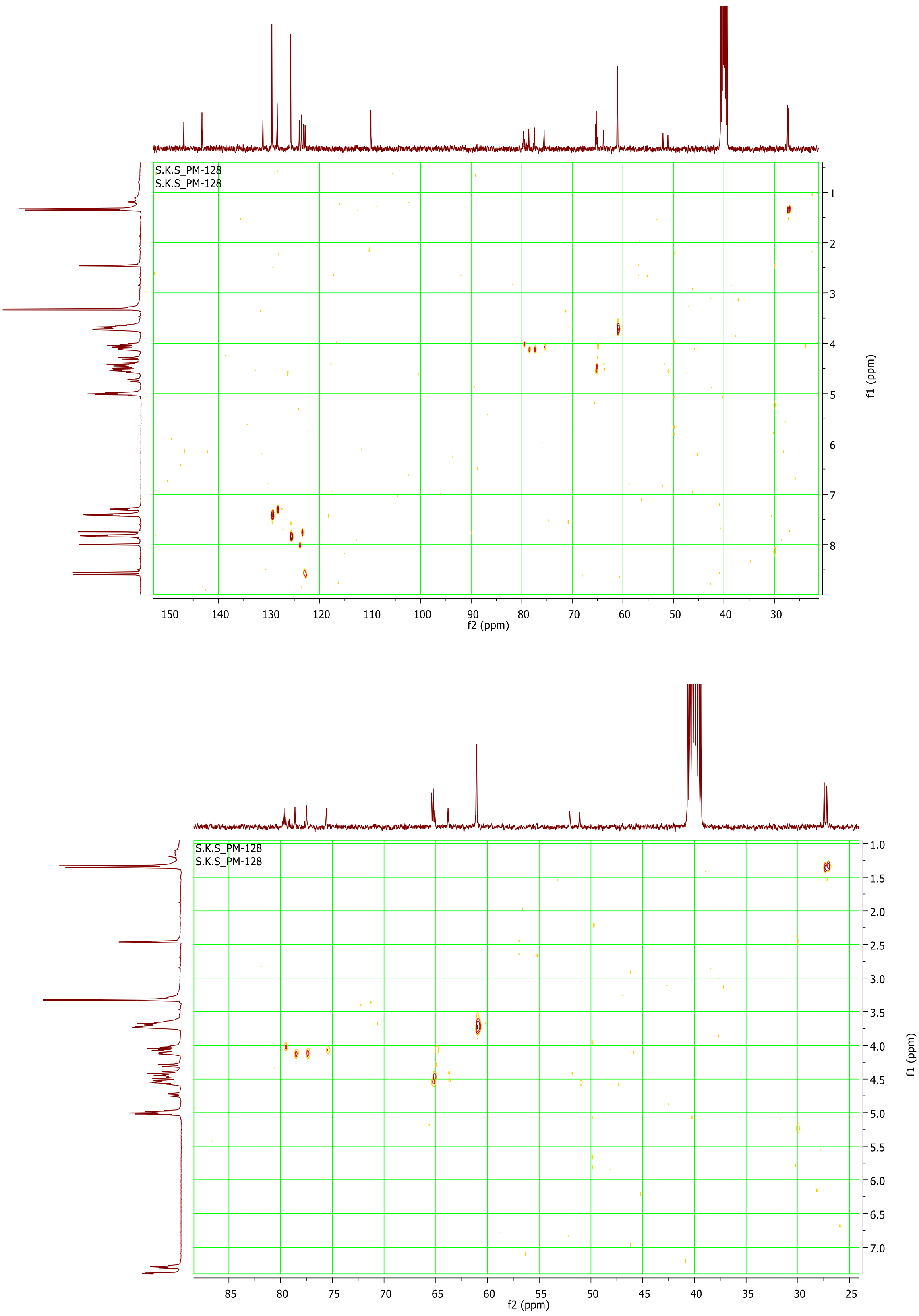

Figure S11. [2]D HETCOR spectrum of compound **20** in DMSO-d_6.

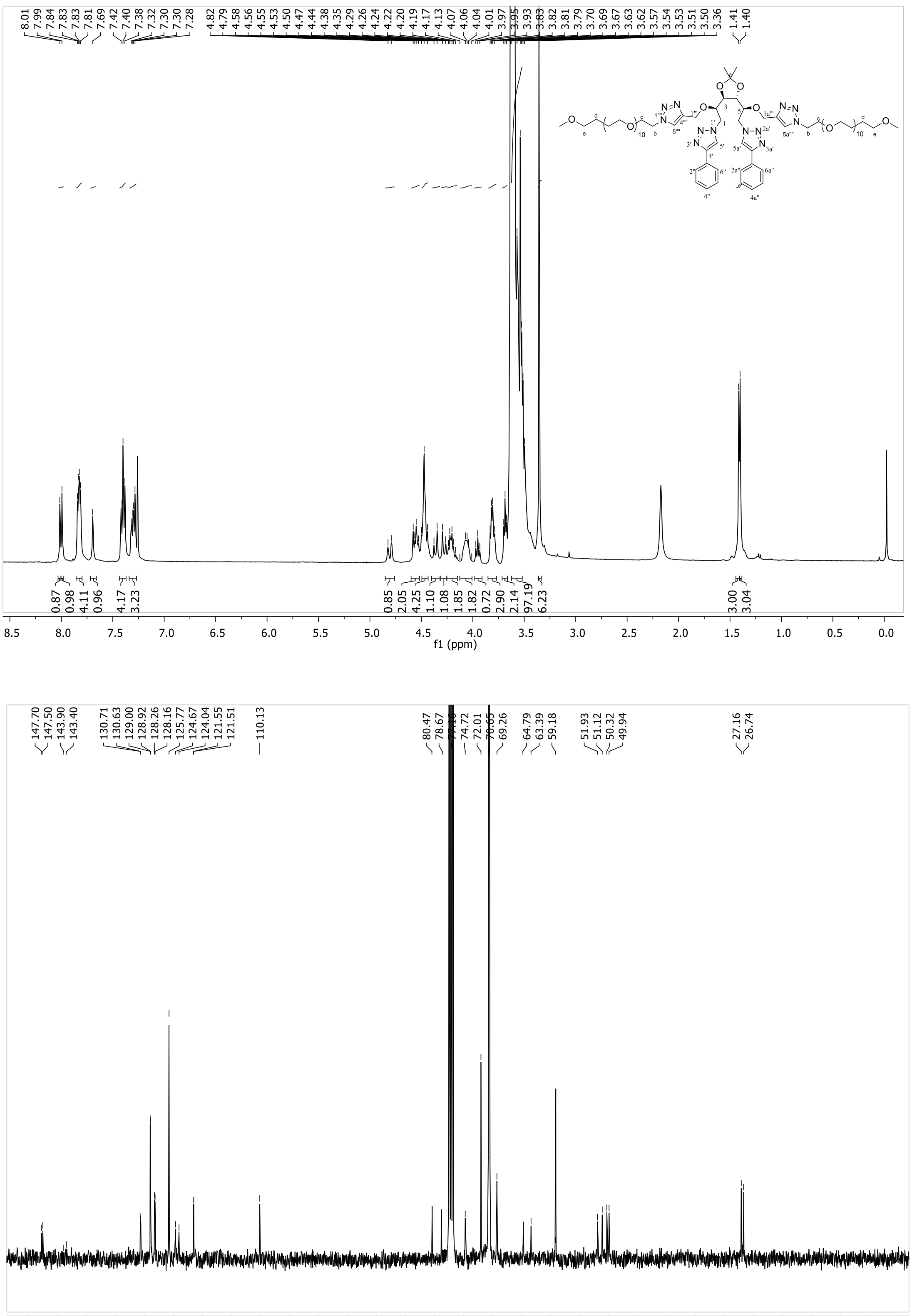

Figure S12. ^{1}H and ^{13}C NMR spectra of compound **21**.

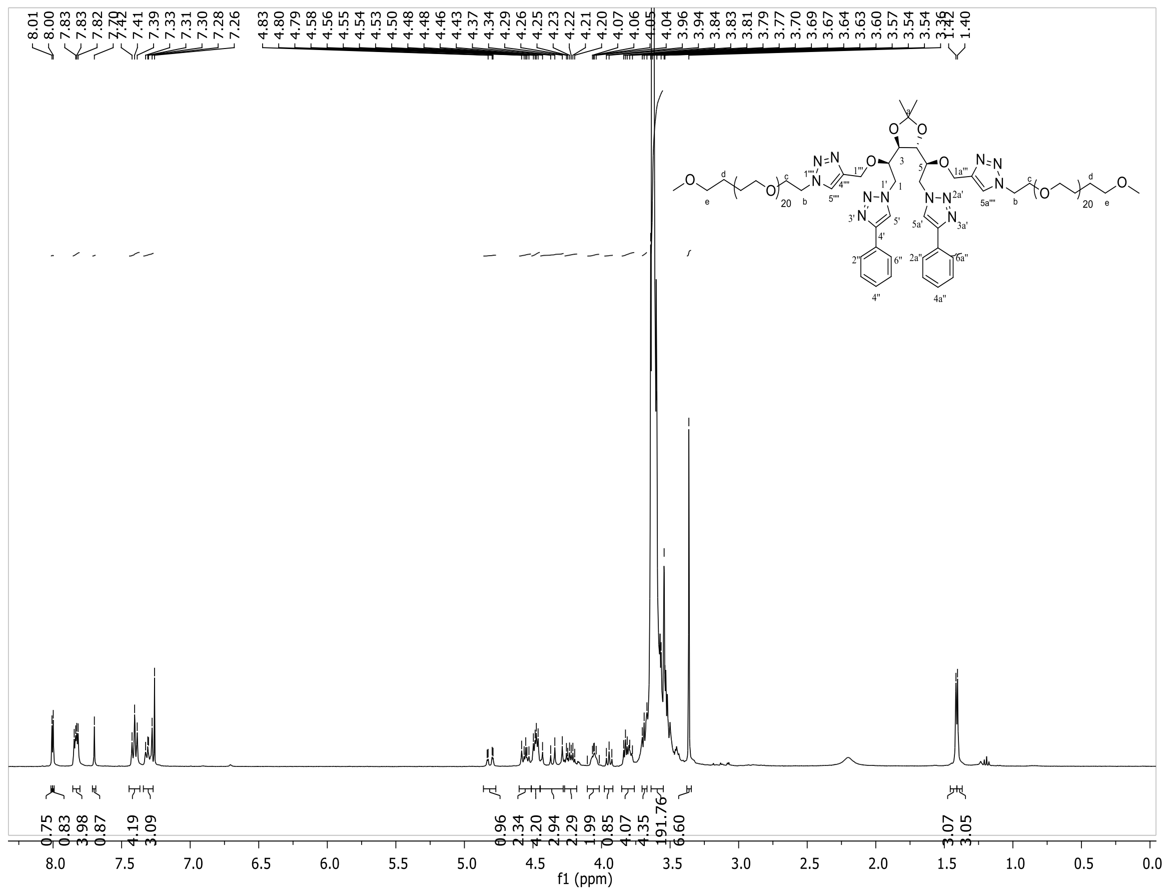

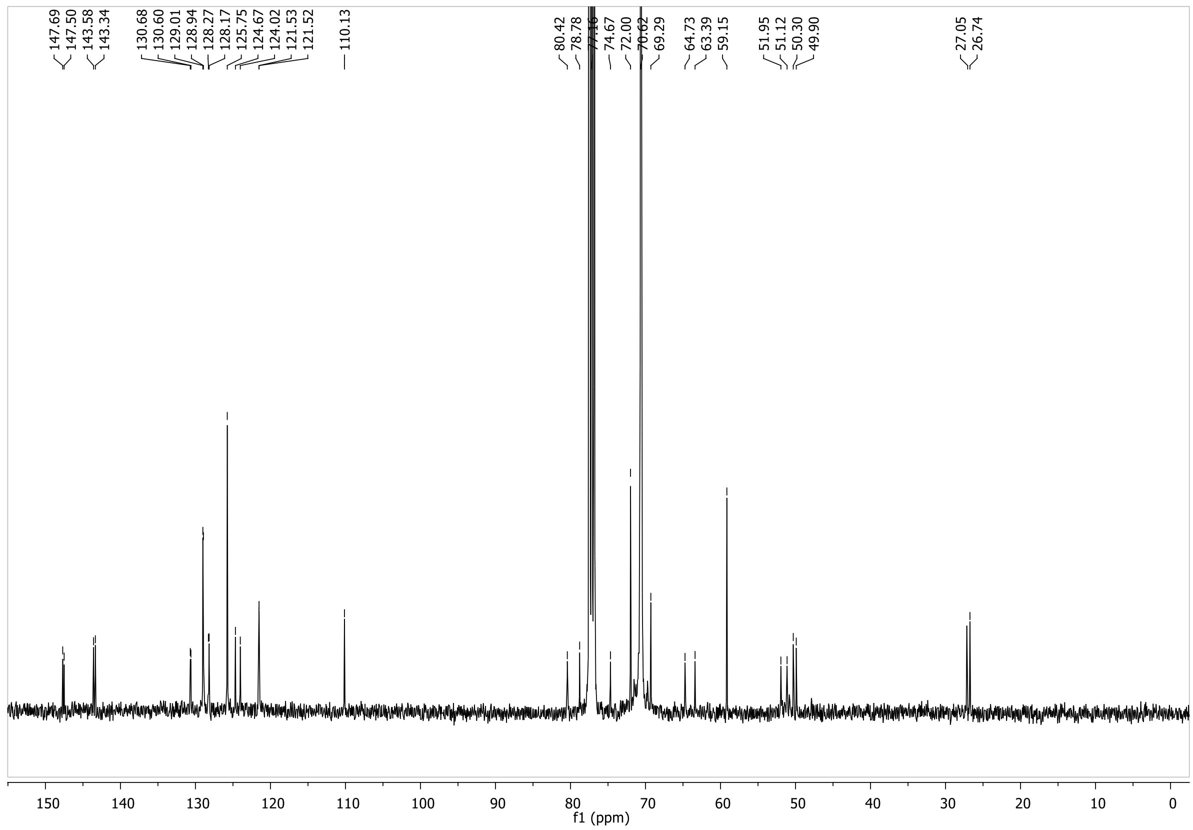

Figure S13. ^{1}H and ^{13}C NMR spectra of compound **22**.

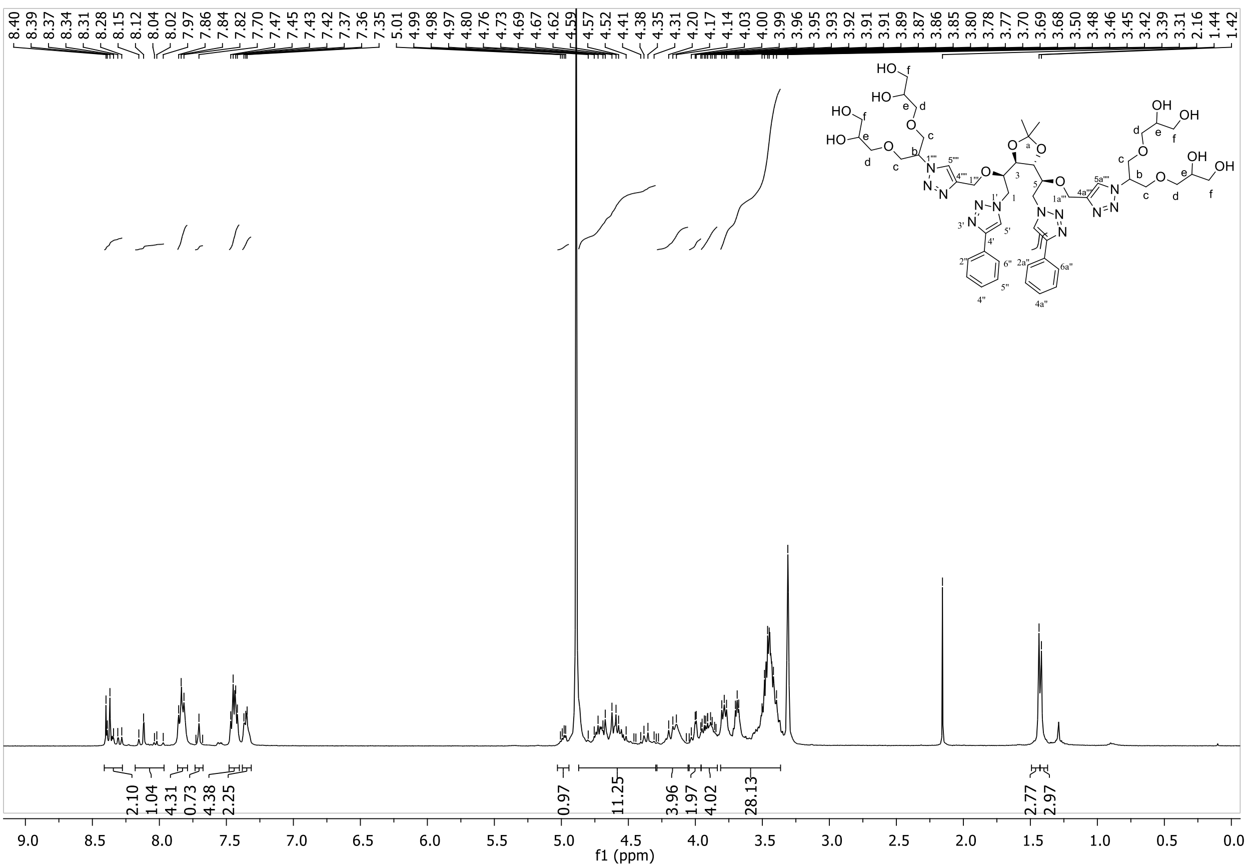

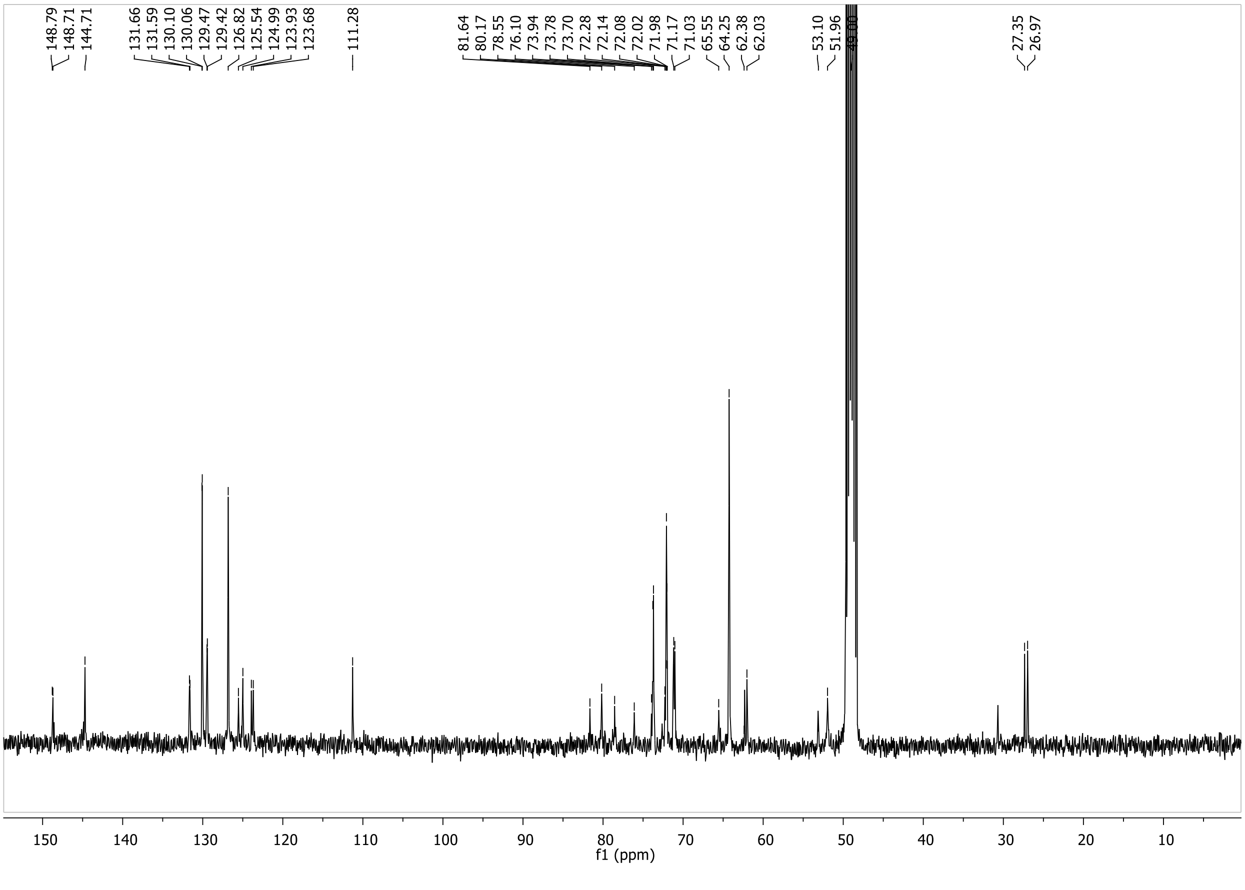

Figure S14. ^{1}H and ^{13}C NMR spectra of compound **23**.

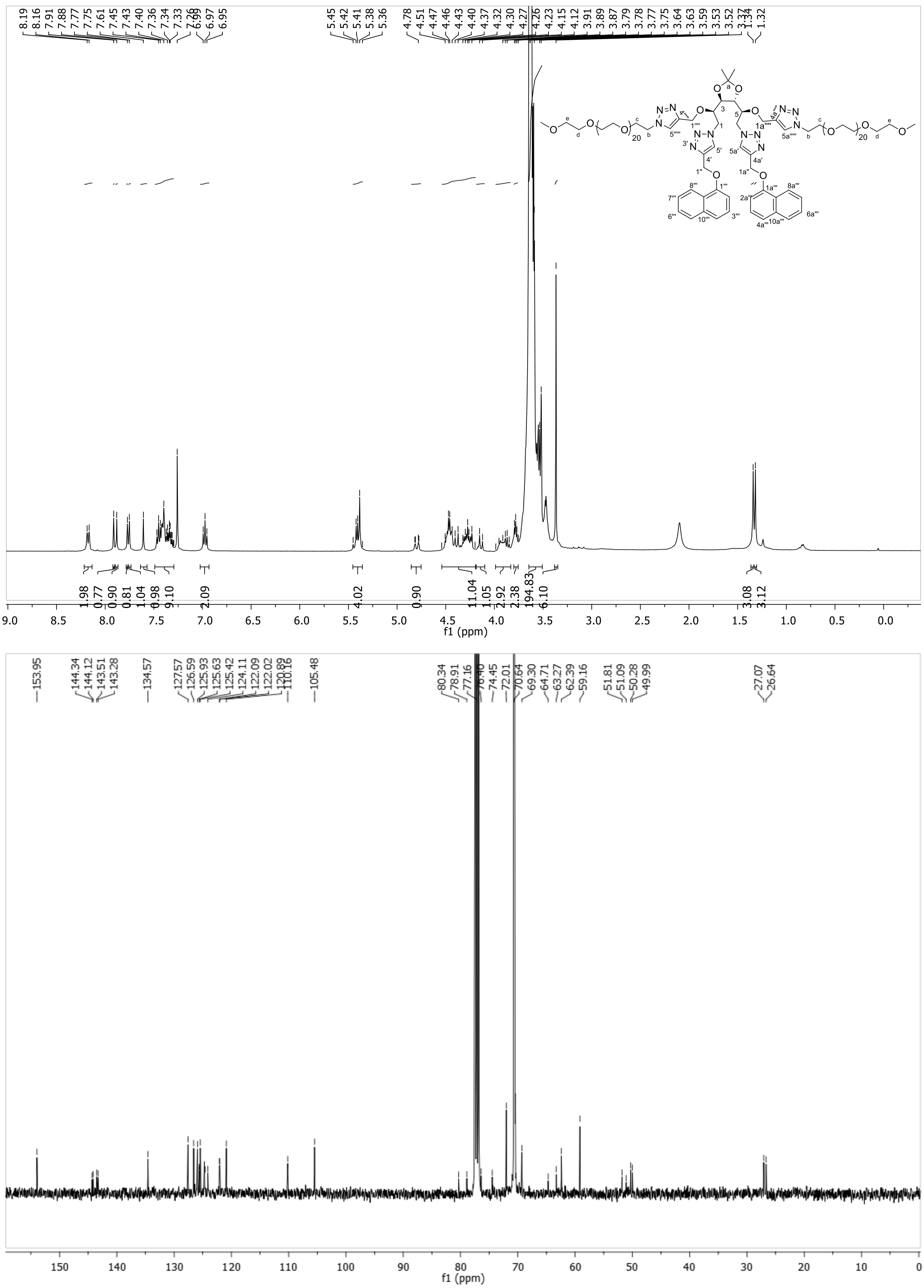

Figure S15. ^{1}H and ^{13}C NMR spectra of compound **24**.

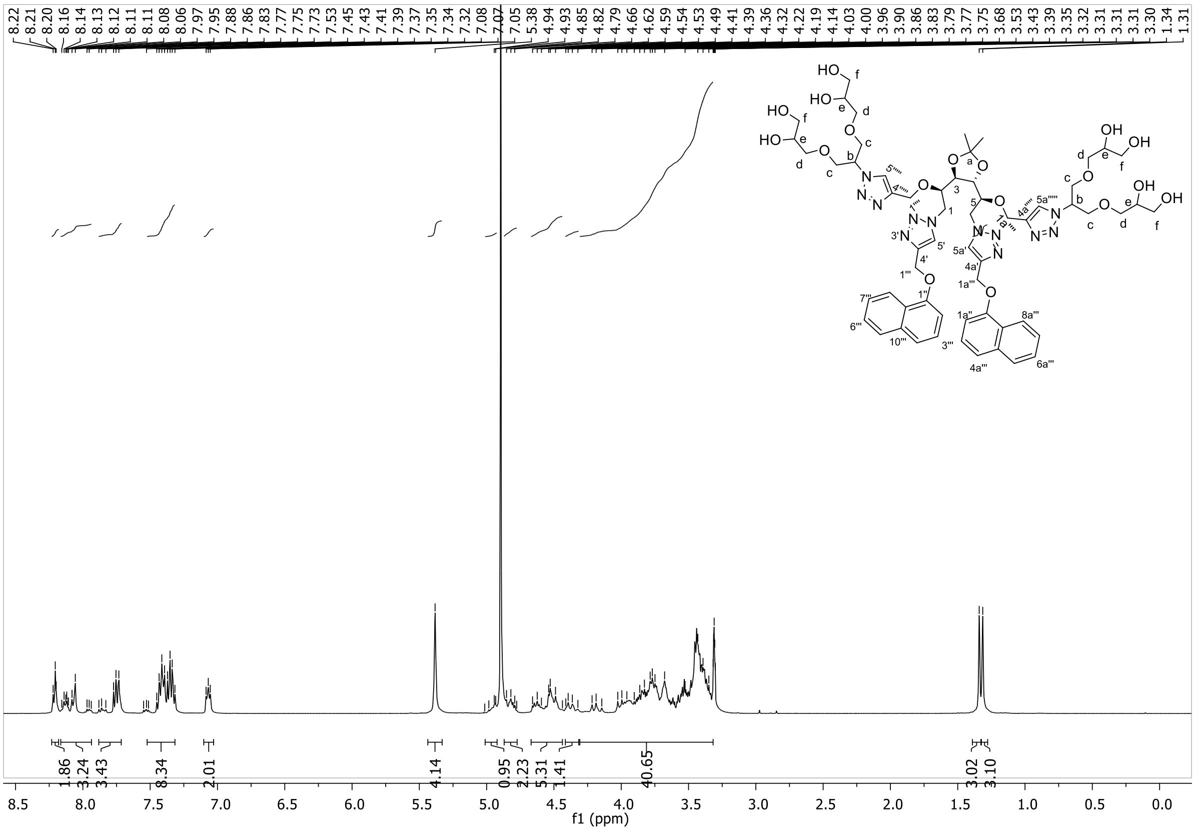

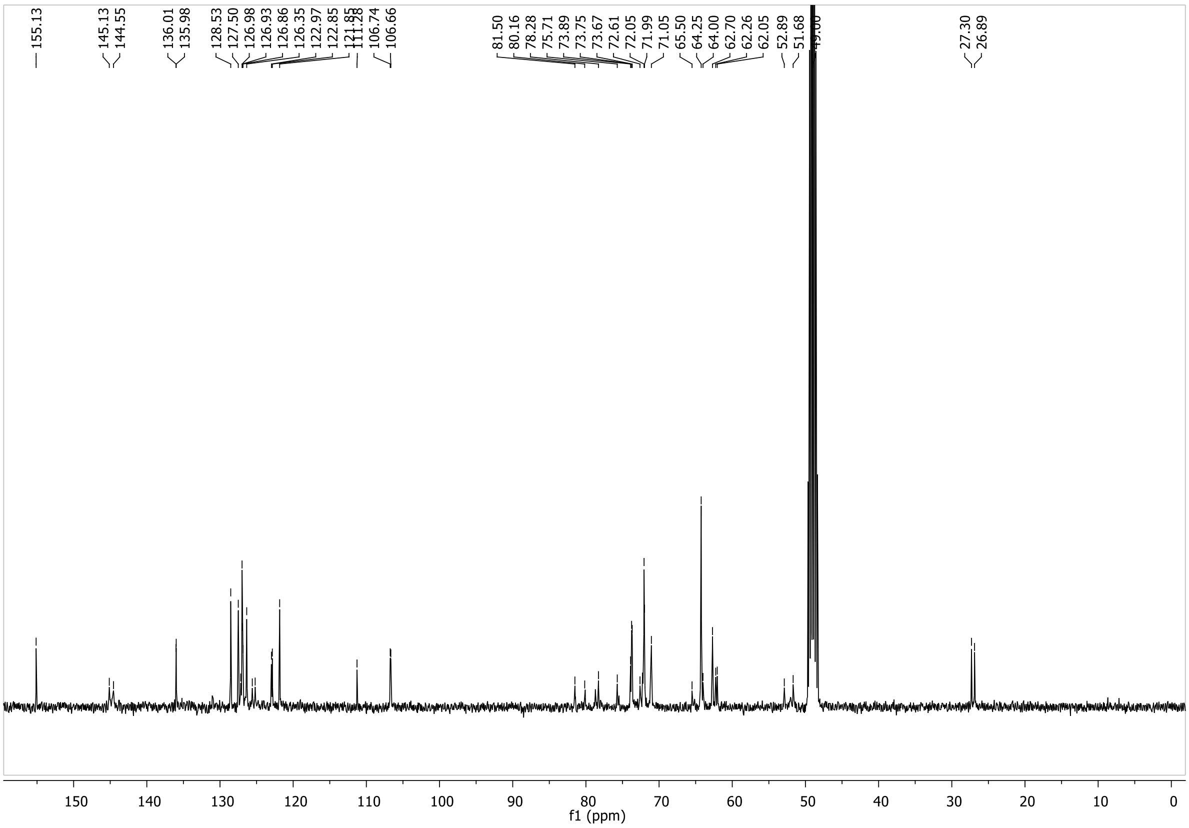

Figure S16. ^{1}H and ^{13}C NMR spectra of compound **25**.

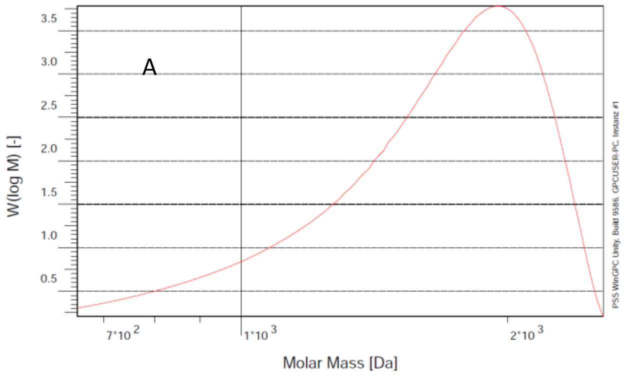

GPC (THF, 1 mL min^{-1}): $\overline{M}_w$ = 1651 g mol^{-1}, $\overline{M}_n$ = 1508 g mol^{-1}, $\overline{M}_z$ = 1769 g mol^{-1}, PDI = 1.09.

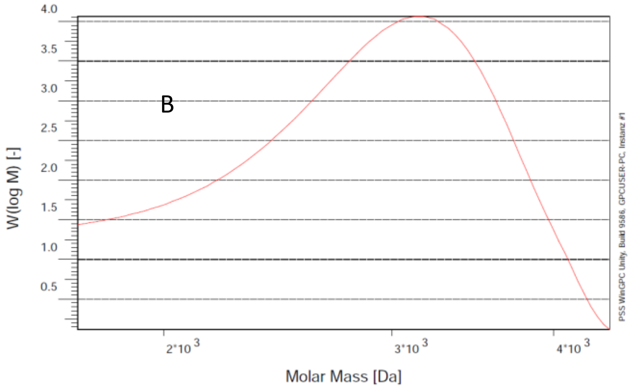

GPC (THF, 1 mL min^{-1}): $\overline{M}_w$ = 2861 g mol^{-1}, $\overline{M}_n$ = 2722 g mol^{-1}, $\overline{M}_z$ = 2995 g mol^{-1}, PDI = 1.05.

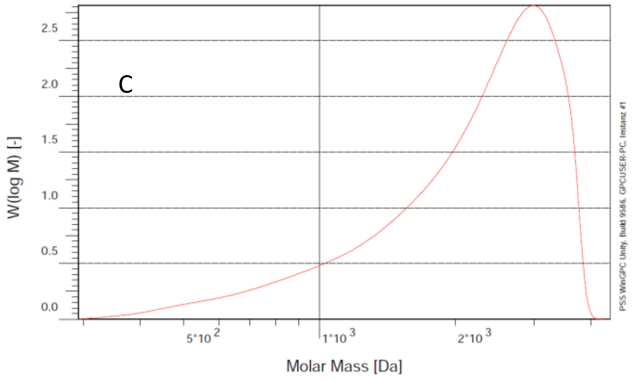

GPC (THF, 1 mL min^{-1}): $\overline{M}_w$ = 2278 g mol^{-1}, $\overline{M}_n$ = 1800 g mol^{-1}, $\overline{M}_z$ = 2606 g mol^{-1}, PDI = 1.26.

Figure **S17**. Gel permeation chromatogram of amphiphiles (A) **21** (B) **22** (C) **24**.

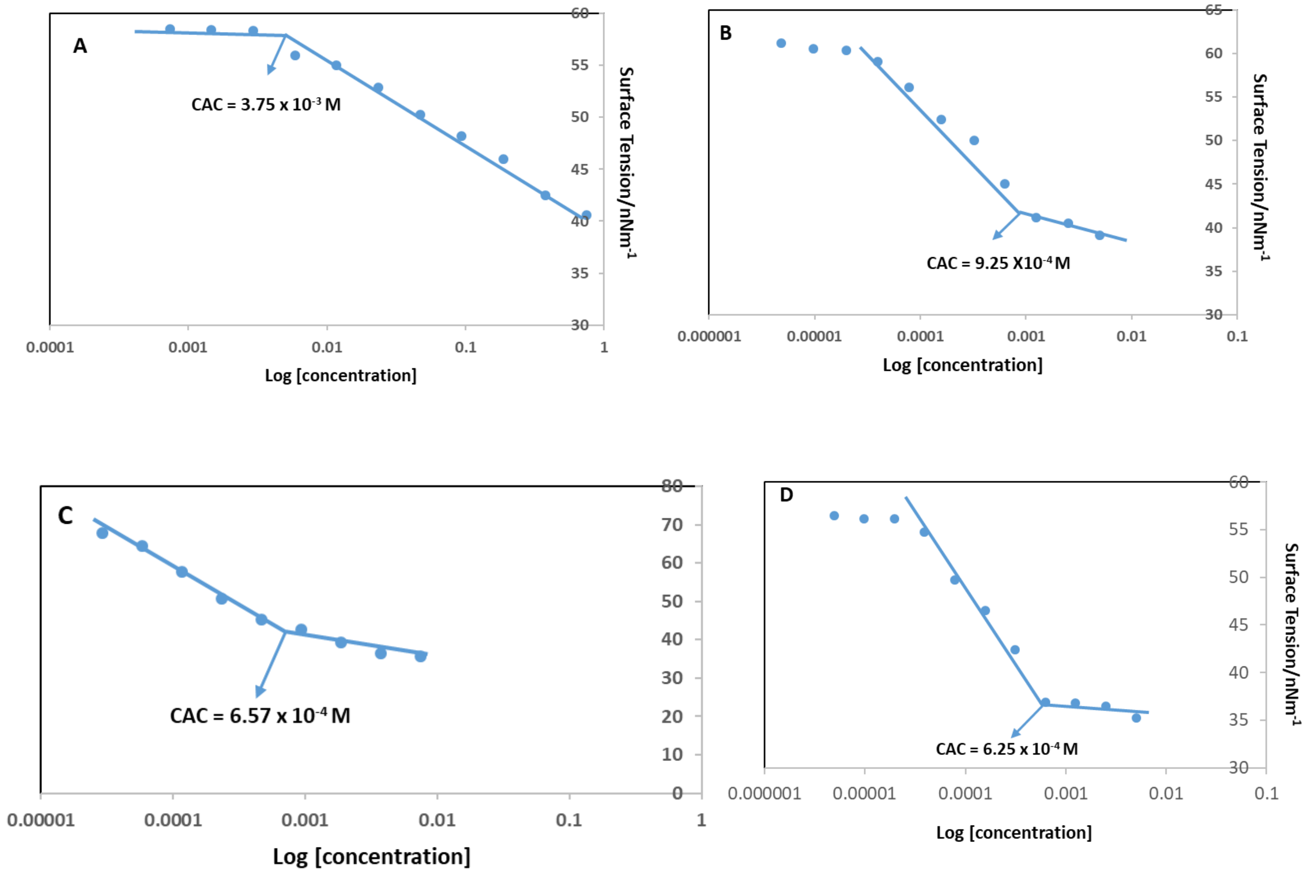

Figure S18. Critical aggregation concentration (CAC) of amphiphiles (A) **21** (B) **22** (C) **23** (D) **25** in aqueous solution by surface tension measurements at 25 ºC.

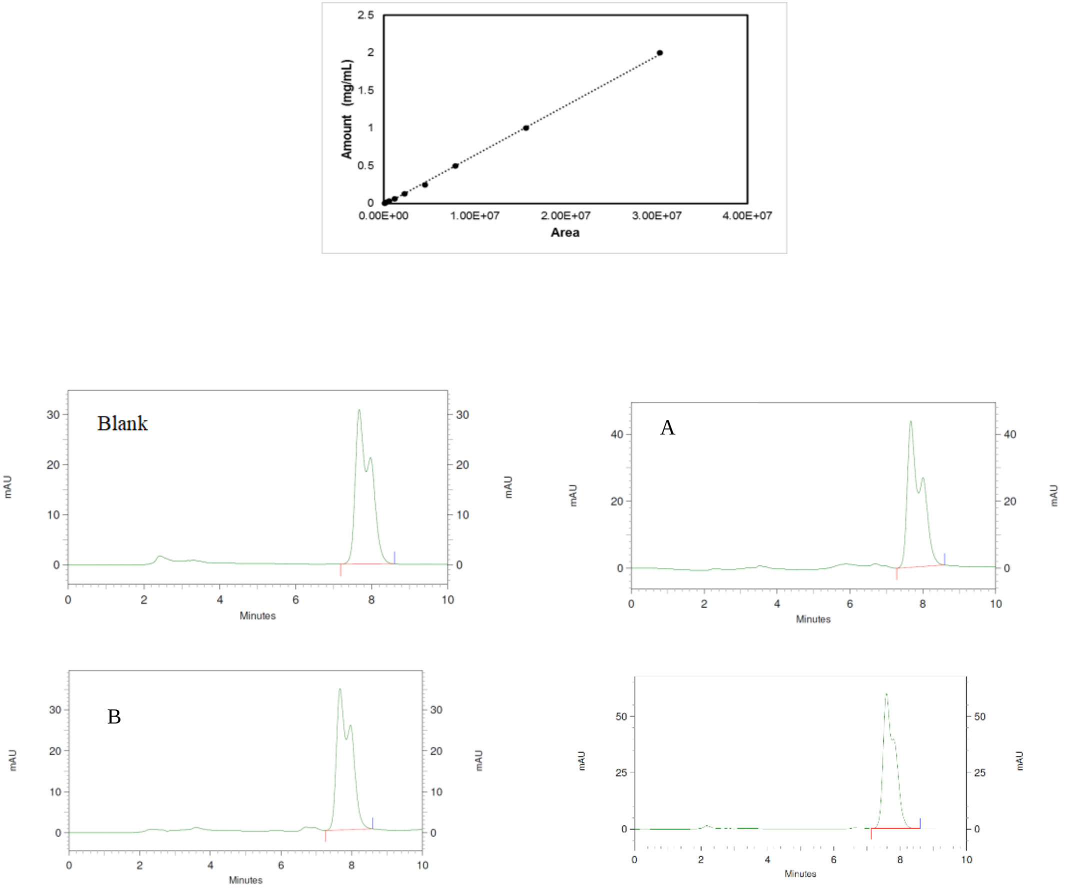

Figure S19. Calibration curve of Dexamethasone using HPLC and representative HPLC chromatogram of dexamethasone encapsulated samples (A) 21(B) 22 (C) 24

References:

1. Gupta, S.; Schade, B.; Kumar, S.; Böttcher, C.; Sharma, S.K.; Haag, R. Non-ionic Dendronized Multiamphiphilic Polymers as Nanocarriers for Biomedical Applications. *R. Small* **2013**, *9*, 894-904.
